# Synthetic strategies of heterocycle-integrated pyridopyrimidine scaffolds supported by nano-catalysts

**DOI:** 10.1039/d3ra00922j

**Published:** 2023-04-14

**Authors:** Mohamed M. Hammouda, Marwa M. Rashed, Khaled M. Elattar, Amany M. A. Osman

**Affiliations:** a Department of Chemistry, College of Science and Humanities in Al-Kharj, Prince Sattam Bin Abdulaziz University Al-Kharj 11942 Saudi Arabia; b Chemistry Department, Faculty of Science, Mansoura University El-Gomhoria Street Mansoura 35516 Egypt elmhammouda@mans.edu.eg; c Toxicology Department, Mansoura Hospital, Faculty of Medicine, Mansoura University El-Gomhoria Street Mansoura 35516 Egypt marwarashed@mans.edu.eg; d Unit of Genetic Engineering and Biotechnology, Faculty of Science, Mansoura University El-Gomhoria Street Mansoura 35516 Egypt khaledelattar2@yahoo.com +20-1010655354; e Chemistry Department, Faculty of Science, Menoufia University Shebin El-Koam Egypt amanyosman812@gmail.com

## Abstract

Nano-catalysts are of special character for the synthesis of organic molecules with high efficiency, and exceptional physicochemical properties. The objective of this study was to present an overview of the literature reports concerning the synthetic strategies supported by nano-catalysts and the biological features of heterocycle-integrated pyridopyrimidine scaffolds. The basic topics include the strategies that were adopted to prepare pyrido[2,3-*d*]pyrimidines and pyrido[1,2-*a*]pyrimidines. The synthesis of pyrido[2,3-*d*]pyrimidines was attained through two-, three-, or four-component reactions. The synthesis of spirocyclic systems, including spiro[indoline-pyridopyrimidine] derivatives and arylation reactions, was investigated. The anticipated mechanisms of the diverse target products, in addition to the preparation of the nanocatalysts, were scrutinized. The privileged antimicrobial characteristics, challenges, literature overview, and future prospectives were consistently investigated.

## Abstract 2

The current review explores the scope of the synthetic strategies supported by nano-catalysts and biological aspects of heterocycles-integrated pyridopyrimidine scaffolds.

## Graphical abstract

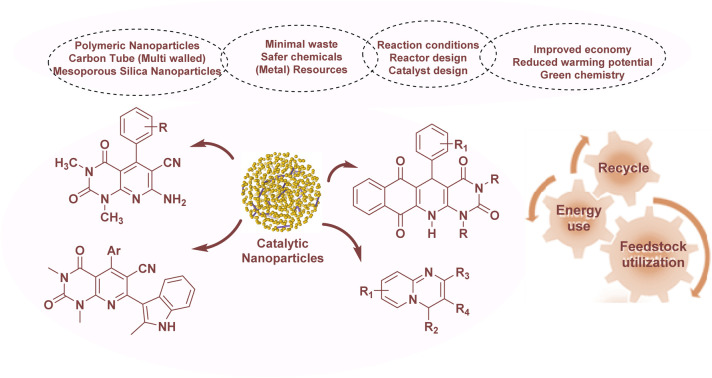

## 1 Introduction

Nanomaterial applications have gained significant interests beyond materials science, including biomedical, chemical, and electronics areas, owing to their extreme superficial area, volume ratios, conductivity, magnetic liability, and catalytic features.^1,2^ In recent times, magnetic nanoparticles have attracted increasing attention due to their exceptional properties and prospective usage in diverse areas. As heterogeneous catalysts, they have generated interest in the synthetic organic field owing to the ease of product isolation, green procedure, recyclability, economics, and simplicity of preparation.^2,3^ Magnetic nanoparticles have likewise acquired interest as feasible ecologically beneficial alternatives of the usual Lewis base, and acid catalysts in numerous synthetic procedures.^4^

Magnetic nanoparticles as solid acid catalysts have emerged as imperative efficient materials in industrial developement. Mainly, for organic reactions, in which water is elaborated, merely numerous solid acids display satisfactory performance. The progress of novel solid acids is predictable to have a key influence on industrial applications in addition to fundamental research. This challenge could be dealt with by manipulative diverse Brønsted acids (SO_3_H, HClO_4_, HBF_4_) on γ-Fe_2_O_3_@SiO_2_,^5–7^ and functionalized hydroxyapatite-encapsulated-γ-Fe_2_O_3_ magnetic nanoparticles.^8–11^ Alternatively, nitrogen-containing heterocycles deliver a significant outlook in natural products and medicinal chemistry. Fused heterocyclic systems integrating a pyrimidine core display vital characters in different biotic and pharmacological developments.^12–20^

Undoubtedly, pyrido[2,3-*d*]pyrimidines are found in various bioactive compounds with bactericidal,^21^ antipyretic,^22^ antitumor,^23^ pharmaceutical,^24^ and antihistaminic^25^ characteristics. Several approaches for the construction of pyrido[2,3-*d*]pyrimidine analogs utilizing 2,6-diaminopyrimidine-4(3*H*)-one,^26,27^ 6-aminouracil, and 6-aminothiouracil have been reported in the last years, which comprised the reactions between α,β-unsaturated carbonyl compounds and 1,3-diketones,^28^ with uracil/or thiouracil and arylidene-malononitriles with 6-aminouracil^29^ or 6-aminothiouracil.^30,31^ Nevertheless, several of these approaches demonstrated difficulties, for instance, low rates of reactions, extraordinarily loaded catalysts, catalyst non-reusability, high temperature, and impurities formation. Several methodologies have been reported for the synthesis of pyridopyrimidines involving the use of nano-catalysts, and in due courses, supportable approaches for the synthesis of privileged bioactive molecules.

**Fig. 1:**
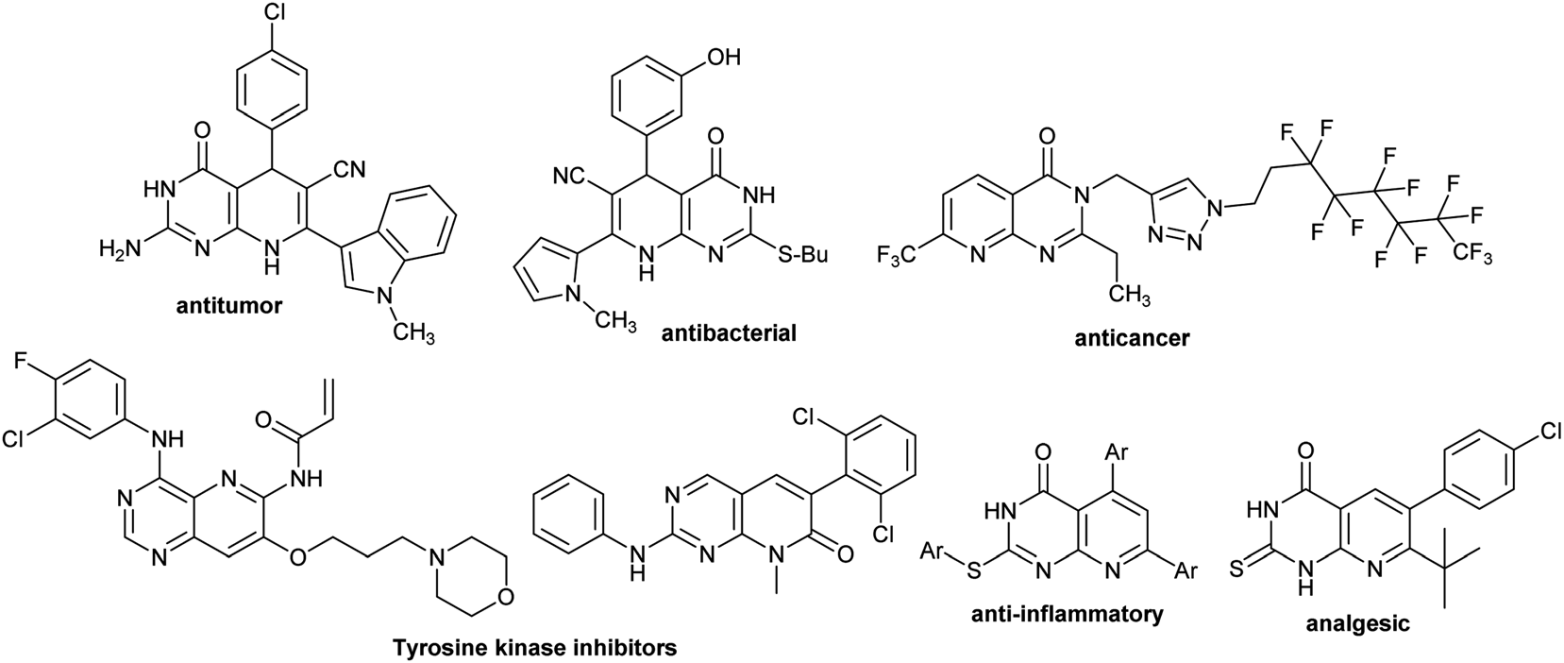
Structures of bioactive molecules integrated pyridopyrimidine motif.

Heterocycles integrated pyrido[2,3-*d*]pyrimidine verified substantial biological features and are found in the basic skeleton of many drugs (Fig. 1).^32–34^ Diverse scales of biological potency were reported for this class of compounds, for instance, antibacterial,^35^ apoptosis inducers,^36^ antitumor,^37^ anti-proliferative CDK2 inhibitors,^38^ fungicidal,^39^ antihypertensive,^40^ antimalarial,^41^ bronchodilators,^42^ cardiotonic,^43^ analgesic,^44^ antileishmanial,^45^ antifolate agents,^46^ and EGFR inhibitory activity.^47^ Also, pyridopyrimidines generally embrace the excessive potential in the pharmaceutical science field owing to the incorporation of their skeleton in many drugs. Many potential characters were reported for pyridopyrimidines, including antiviral,^48,49^ antimicrobial,^50^ antihypertensive, anti-tumor,^51^ antihistaminic,^52^ antimalarial, protein kinase inhibitors,^53^ diarrhea treatment,^54^ anti-inflammatory and analgesic activities,^55^ besides other medicinal features.^56^

Rostamizadeh *et al.*^57^ have utilized (Fe_2_O_3_)-MCM-41-*n*PrNH_2_ nanocatalyst in a green technique for the preparation of 2-amino-phenyl pyrido[4,3-*d*]pyrimidines applying two-component reactions. Thus, reactions of (*E*)-3,5-bis(benzylidene)-4-piperidones with guanidine carbonate under magnetically recoverable nanocatalyst and solvent-free conditions yielded the desired products, pyrido[4,3-*d*]pyrimidines. The objective of the present review is to highlight the diverse nano-catalysts applied to prepare heterocycles integrated pyridopyrimidine core. Through our preceding work regarding the significance of reactive synthons in the synthesis of many heterocycles,^58,59^ and many reviews that deliberated the chemistry fused pyrimidines,^60–67^ and pyridopyrimidines,^68–70^ we herein highlight the advancements in the synthetic strategies of heterocycle-integrated pyridopyrimidine scaffolds supported in nano-catalysis. The theme of the research is apprehensive with reviewing the mechanisms of the diverse reactions, nanocatalyst preparation, the benefit of the used nanocatalyst, the nanocatalyst's efficient role for improved product yields, and increased reaction rates.

## 2 Synthesis of annulated pyrido[2,3-*d*]pyrimidines

### 2.1 Two-component reactions

**Scheme 1:**
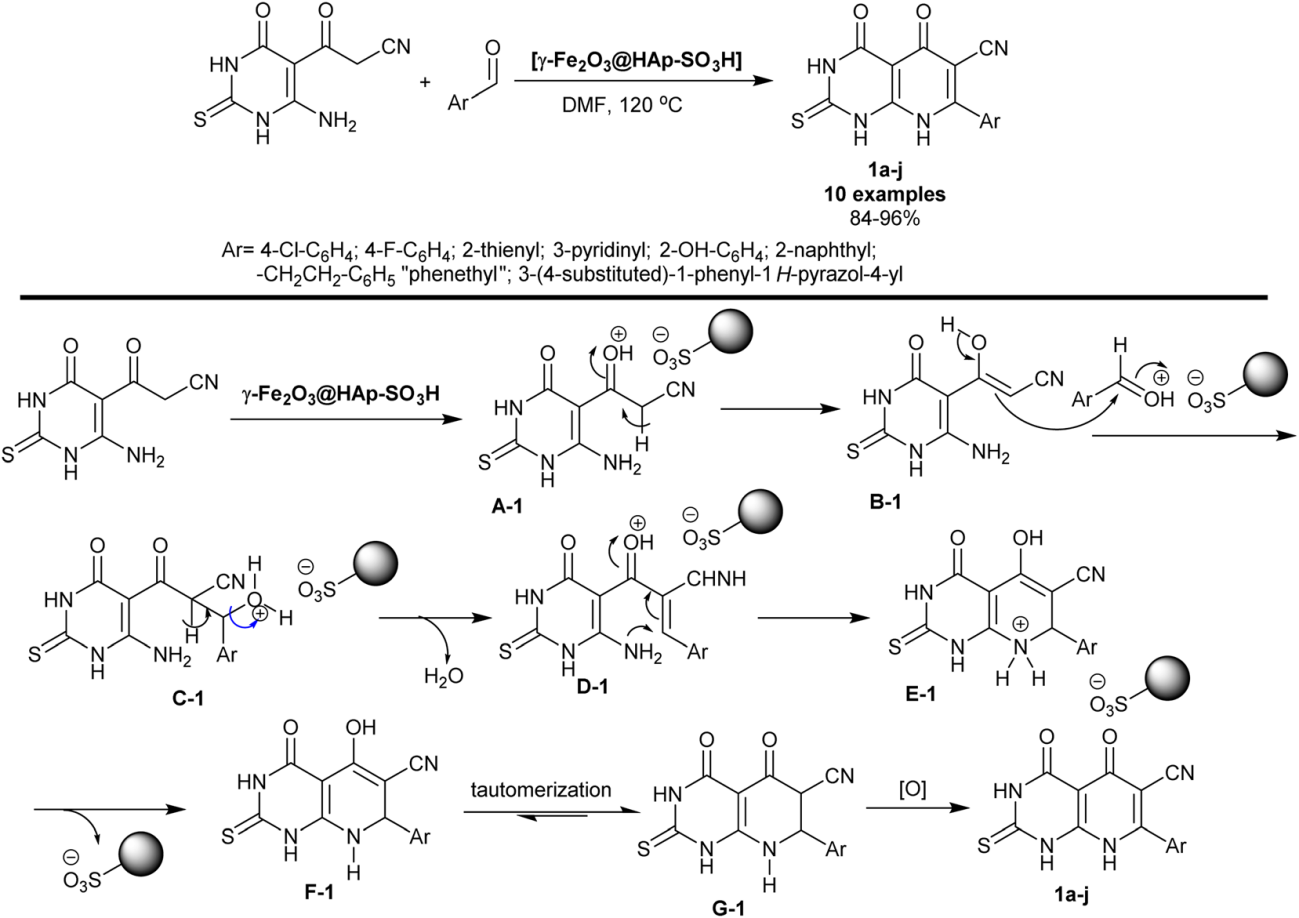
Synthesis and the plausible mechanism of hexahydropyridopyrimidines.

Pyridopyrimidines signify an exceedingly imperative category of compounds that reveal an extensive scale of biological features. Metal oxide catalysts in the nanoscale have been widely considered for their application in organic reactions due to their exceptional landscapes, for instance, high surface area and hole sizes as promotions. Mamaghani *et al.*^71^ have also utilized the [γ-Fe_2_O_3_@HAp-SO_3_H] nanocatalyst in the synthesis of hexahydropyrido[2,3-*d*]pyrimidines 1a–j. The procedure involved the reactions of aryl aldehydes with cyanoacetyl-pyrimidinone in DMF under heating and nanocatalysis conditions. The bicyclic products were acquired in good yields (84–96%) owing to the nanocatalyst role and depending on the nature of the structured substituents on the benzene ring of the aldehyde (Scheme 1).

The reaction sequence, as depicted in Scheme 1, proceeded through the initial protonation of the carbonyl group of the caynoacetyl moiety by the action of the nanocatalyst that accelerates the enolization step. The condensation of the aryl aldehydes that were activated by the nanocatalyst with the enolized cyanoacetyl intermediate generated the arylidene intermediates D-1. Intramolecular cyclization of the intermediate D-1 generated the intermediate E-1. The nanocatalyst was released from the reaction followed by enol-keto tautomerization and oxidation to give the final target bicyclic products 1a–j. The two substrates reacted to give the bicyclic products in one step through the formation of new C=C and C–N bonds.^71^

**Scheme 2:**
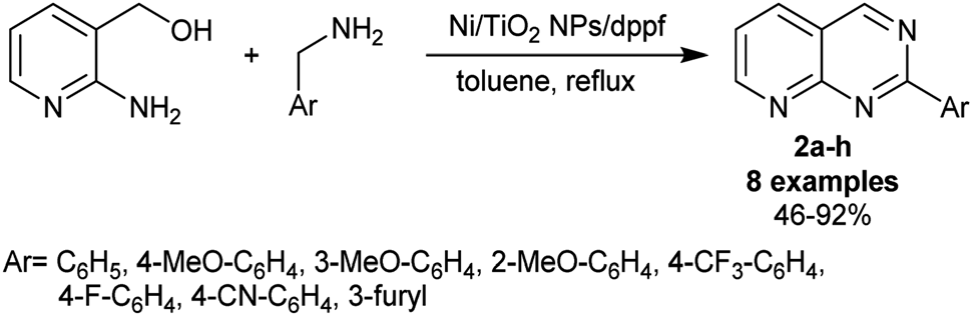
Synthesis of 2-aryl-pyrido[2,3-*d*]pyrimidines.

Titanium dioxide has been reported as an efficient nano-catalyst, for instance, as catalysis in the shift of water gas,^72^ hydrodesulphurization,^73^ dehydrogenation,^74,75^ and thermal decomposition.^76^ A simple procedure was applied by Kaiba *et al.*^77^ for the synthesis of 2-aryl-pyrido[2,3-*d*]pyrimidines 2a–h using Ni-doped TiO_2_ nanoparticles. Thus, two-component reactions of (2-aminopyridin-3-yl)methanol with aryl methan-amines in toluene at their reflux temperature under the optimized conditions yielded the desired pyridopyrimidines 2a–h (Scheme 2). In particular, dppf was used as a ligand, and toluene is preferred over other solvents such as DMSO, THF, acetone, chloroform, and DMF to improve the product yield. A sol–gel procedure was used for the preparation of the nanoparticles of Ni-doped TiO_2_ by the treatment of titanate solution in ethanol with nickel chloride and the subsequent addition of an aqueous solution of hydrochloric acid. The mixture was kept under sonication conditions and incubated for four days at 40 °C for complete hydrolysis. The procedure presented an exceptional yield (up to 92%). The substituents at the phenyl ring of the aryl amines affected the product yields, in which the fluoro, trifluoromethyl, and cyano substituents at the *para* position produced moderate yields (59–68%), while the incorporation of furan-3-ylmethanamine as the aryl amine substrate presented the lowest yield (46%).

**Scheme 3:**
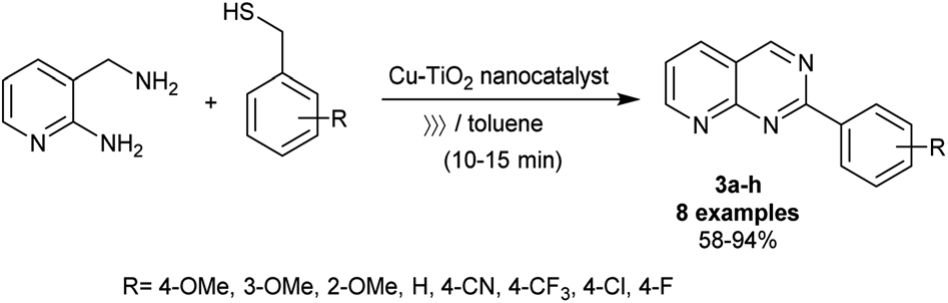
Synthesis of 2-arylpyrido[2,3-*d*]pyrimidines.

Recently, Dahi-Azar *et al.*^78^ reported an impressive procedure for the synthesis of 1,3-dimethyl-2,4-dioxo-5-phenyl-1,2,3,4,5,8-hexahydropyrido[2,3-*d*]pyrimidine-7-carboxylic acids in 96–98% yields using a recyclable nanocatalyst. Thus, cyclocondensation reaction of 4-substituted phenylmethylidenepyruvic acids with 6-amino-1,3-dimethyluracil under ethanol drop grinding, and nano-catalytic conditions using cadmium oxide nanoparticles at room temperature. The procedure provided ease of the product's workup, shortened reaction times, simplicity of the preparation, and improved product yields. Geesi *et al.*^79^ have recently also established the synthesis of 2-arylpyrido[2,3-*d*]pyrimidines 3a–h*via* the reactions of 3-(aminomethyl)pyridin-2-amine with aryl methanethiol derivatives under ultrasonic irradiation conditions (Scheme 3). In this route, Cu-doped TiO_2_ nanoparticles were applied as an efficient nanocatalyst under ambient conditions. Particularly, the nanocatalyst enables the pyrimidine ring cyclization with the release of hydrogen sulfide molecules. The free-catalyst conditions in toluene containing TPP as a ligand at room temperature led to no reactions. The best yield was obtained from the reactions of 3-(aminomethyl)pyridin-2-amine with various heteroarylthiol derivatives under optimized conditions using toluene as a solvent, nano-Cu-doped TiO_2_ as a catalyst, and TPP (10 mol%) as a ligand. In this reaction type, the alkyl substituents at the phenyl ring are preferred for improving the yields of the products, whereas the nitrile substituent at the para position of the phenyl ring presented the minimum yield of the product (58%). The best yield (94%) was acquired for the methoxy substituent at the para position, while the methoxy substituent at the meta and ortho positions led to diminished product yields owing to the steric hindrance factor.

**Fig. 2:**
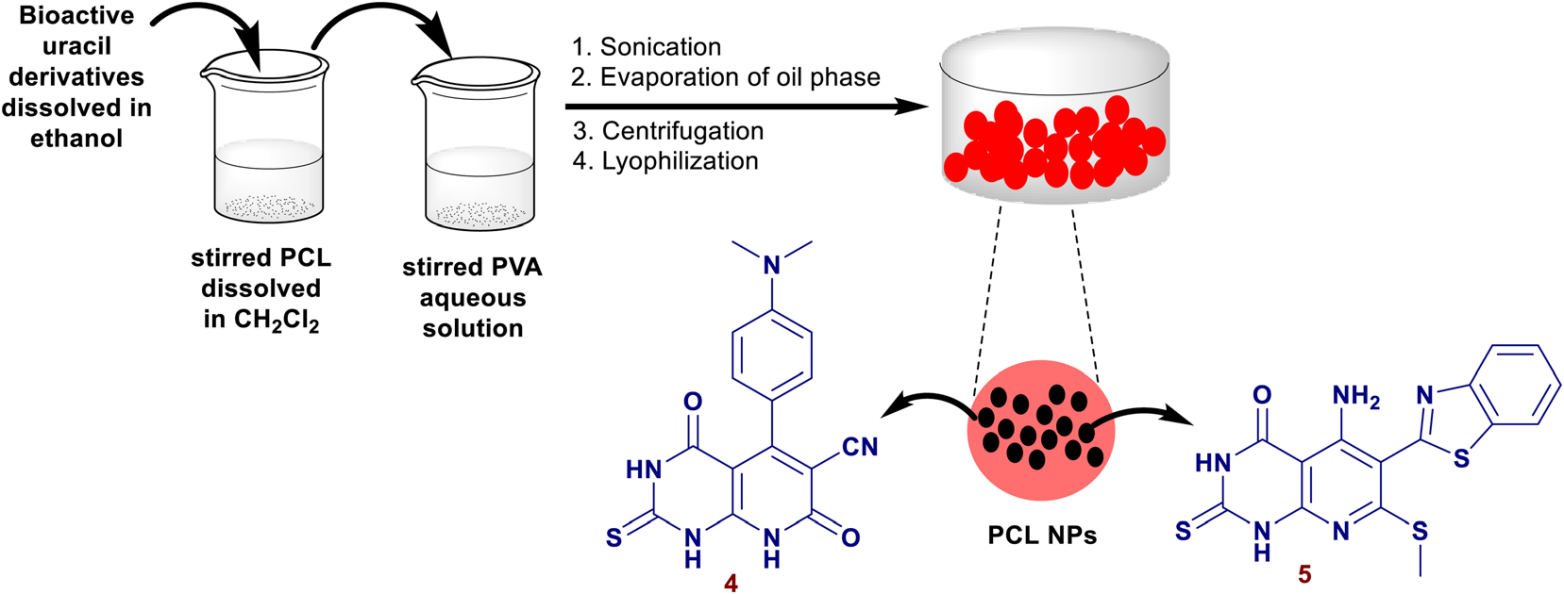
The nanoencapsulation process of pyridopyrimidines.

Fadda *el al*.^80^ have synthesized a series of pyrido[2,3-*d*]pyrimidines by reactions of 6-amino-2-thiouracil with cyanopyridine or enaminonitriles or arylidenes or ethyl 3-chloro-pyridazine-4-carboxylate or 1-(3-chloro-pyridazin-4-yl)ethan-1-one or substituted 4-chloro-pyrimidine-5-carbonitriles in the absence of nano-catalyst. Two compounds from this series *e.g.* pyridopyrimidines 4, and 5 (Fig. 2) were unified into polycaprolactone nanoparticles by a single emulsion-solvent evaporation performance. The technique provided the encapsulation of hydrophobic ingredients, for instance, the entitled compounds 4 and 5, as these compounds are insoluble in water and the nanoencapsulation improves their bioactivity by increasing their solubility. The nanoencapsulation also provided a stable colloidal dispersion at the higher concentrations of the sample leading to amended bioavailability. The unmodified compounds were *in vitro* appraised as antimicrobial agents against varied Gram-positive, Gram-negative, and fungal species. The modified pyridopyrimidines 4 and 5 revealed potent activities against *B. subtilis* (3.125 μg mL^−1^) and *B. thruringiensis* species (6.25 μg mL^−1^). The nanoencapsulation improved the antimicrobial results of both compounds.

### 2.2 Three-component reactions

#### 2.2.1 Reactions involved activated nitriles and ketones

**Scheme 4:**
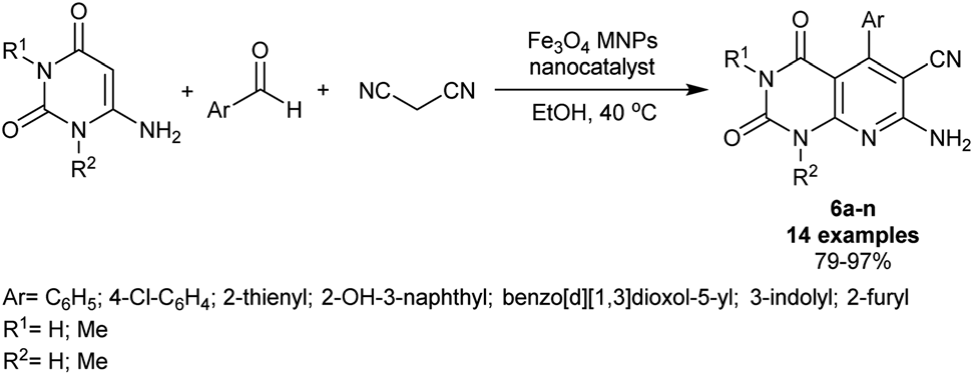
Three-component synthesis of enaminonitriles of tetrahydropyridopyrimidines.

A great literature of varied pyrido[2,3-*d*]pyrimidines was synthetically adopted under nanocatalysis and green conditions. Kidwai *et al.*^4^ have also utilized Fe_3_O_4_ magnetic nanoparticles as a very efficient nanocatalyst for the synthesis of pyridopyrimidine series 6a–n in 79–97% yields. The procedure is an economic, simple, and green protocol that involved a simple preparation of the nanocatalyst, as well as the ease of product separation and purification. Therefore, gently heating the reactants such as disubstituted amino-uracils with aryl aldehydes and malononitrile in a one-pot procedure in ethanol under the ambient conditions gave the desired bicyclic compounds 6a–n (Scheme 4). Consequently, the pyridine ring closure was attained during the reaction mechanistic sequence as mentioned previously. On a large scale of synthesis, the produced enaminonitriles can serve as reactive precursors for the future preparation of various heterocycle-incorporated pyridopyrimidine skeletons. The use of *para*-chloro-benzaldehyde produced the most maximum yield (97%), while aldehydes with 2-furyl and benzo[*d*][1,3]dioxol-5-yl substituent presented the least yields (79–81%) along with improved yields using thiophene-2-carbaldehyde and 1*H*-indole-3-carbaldehyde. Also, the dimethyl substitution and unsubstituted amino-uracil compounds did not show notable impacts on the product yields.

**Scheme 5:**
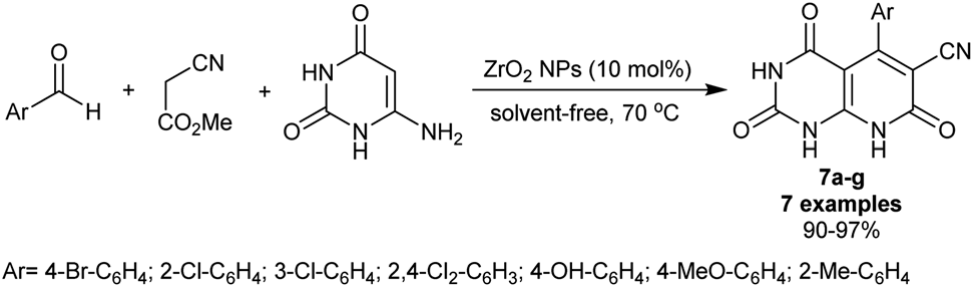
Multicomponent synthesis of trioxo-hexahydropyridopyrimidines.

Abdolmohammadi *et al.*^81^ have synthesized a series of trioxo-hexahydropyrido[2,3-*d*]pyrimidines 7a–g (7 examples) using ZrO_2_ nanoparticles (10 mol%). Subsequently, three-component one-pot reactions of aryl aldehydes with methyl 2-cyanoacetate as an activated nitrile, and 6-amino-uracil under nanocatalytic and solvent-free conditions afforded the bicyclic products in exceptional yields (90–97%) (Scheme 5). The solvent-free conditions provided the green protocol for this type of reaction along with the nanocatalyst efficiency for improved product yields. The reaction sequence was planned through tandem Knoevenagel–Michael addition accompanied by cyclization with the removal of the methanol molecule. The reported mechanism for this work expected that the final step is aromatization although it should be enol-keto tautomerization to yield the bicyclic products 7a–g.

**Scheme 6:**
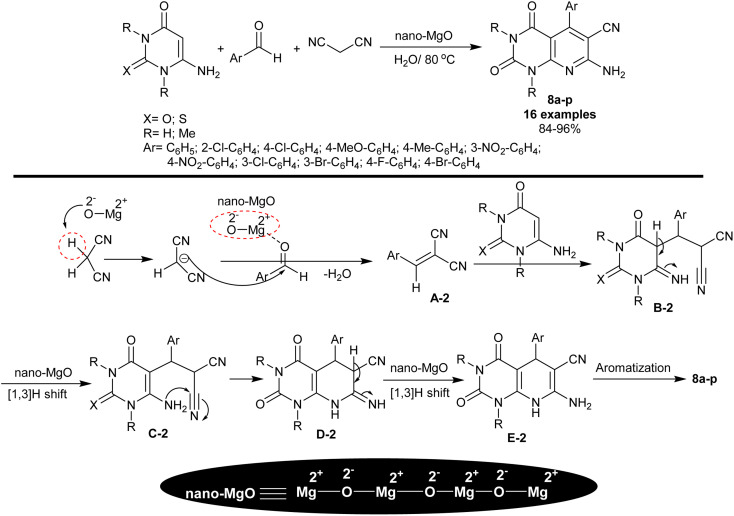
Synthesis of pyrido[2,3-*d*]pyrimidines under catalytic nano-MgO.

In 2015, Rad and Mokhtary^82^ reported the synthesis of enaminonitrile of tetrahydropyrido[2,3-*d*]pyrimidine analogs 8a–p through multicomponent one-pot reactions catalyzed by nano-MgO. Therefore, reactions of 6-amino-uracil/thiouracil or their *N*,*N*-dimethyl derivatives with aryl aldehydes and malononitrile furnished the expected pyrido[2,3-*d*]pyrimidines 8a–p (Scheme 6). The reactions were run in water under heating and nano-catalytic conditions. The procedure has green aspects, in which nano-MgO was applied with simple performance to prepare the target compounds with high efficiency, enhanced yields, and ease of product workup. The method averted the utility of either toxic solvents or catalysts. As shown in Scheme 6, the nano-MgO catalyst activated malononitrile and the aryl aldehydes for condensation to outline the arylidene intermediate A-2. The intermediate A-2 reacted with 6-amino-uracil/thiouracil or their *N*,*N*-dimethyl derivatives through a Michael-addition type to generate the intermediate B-2, which followed rearrangement by [1,3]H transfer to form intermediate C-2. Pyridine ring cyclization was achieved by intramolecular nucleophilic attack of the amino group of the intermediate C-2 at the nitrile group to form intermediate D-2. The nano-MgO enables the tautomerization through [1,3]H transfer to generate the intermediate E-2, and subsequent aromatization to yield the bicyclic products 8a–p. Generally, the Lewis basic and acid sites of the nanocatalyst play a significant character in the activation of substrates for the reaction's increased rate. The *para*-chloro or nitro substituents tended to have the highest yields of the products, whereas the *para*-alkyl or *ortho* substituents tended to reduce product yields.

**Scheme 7:**
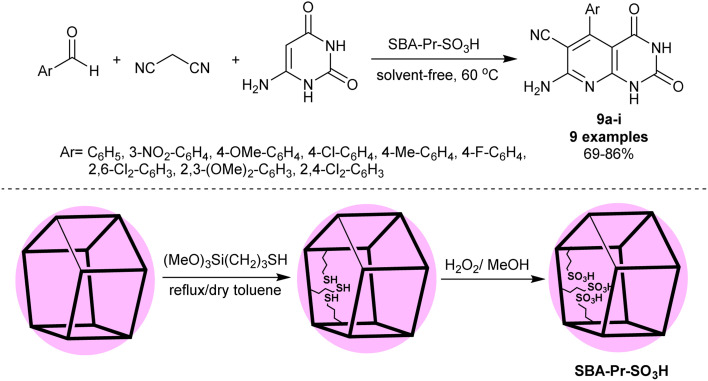
Multicomponent synthesis of pyridopyrimidines under nanocatalyst and free-solvent conditions.

Ziarani *et al.*^83^ have synthesized a series of enaminonitriles with tetrahydropyrido[2,3-*d*]pyrimidine motif 9a–i through nano-catalyzed reactions of 6-aminouracil with activated nitrile, and aryl aldehydes. The reactions were accomplished in a one-pot multicomponent technique through heating under solvent-free conditions. The SBA-15-Pr-SO_3_H nanocatalyst was applied for this purpose in a trial to improve the reaction yields of the previously prepared compounds 9a–i.^84,85^ The nanocatalyst did not provide enhanced product yield in the comparison of the product 9a yield with the previous ZrO_2_ nanoparticles (96%) and TEBAC (97%), but we did not compare the other derivatives in this series. The procedure provided ease of product separation, catalyst reusability, shortened reaction time, and green protocol. The nanocatalyst was prepared by heating 3-(trimethoxysilyl)propane-1-thiol in dry toluene followed by oxidation with hydrogen peroxide to obtain sulfonic acid functionalized SBA-15 (Scheme 7).

The antimicrobial assessment revealed potent activities for the compounds (Scheme 7) with Ar = C_6_H_5_ (28 and 23 mm), 4-ClC_6_H_4_ (26 and 30 mm), 4-CH_3_C_6_H_4_ (28 and 20 mm), 4-FC_6_H_4_ (30 and 24 mm) and 2,6-Cl_2_C_6_H_3_ (28 and 18 mm) along with inactivity for the compounds with 3-NO_2_C_6_H_4_, 4-OCH_3_C_6_H_4_, and 2,3-(OCH_3_)_2_C_6_H_3_ against *B. subtilis* and *S. aureus* species compared to chloramphenicol and gentamicin standards antibiotics. Thus, the introduction of nitro and methoxy substituents in the phenyl ring is not favored for potent antimicrobial consequences. Also, compounds 4-ClC_6_H_4_ (8 and 12 mm) and 4-CH_3_C_6_H_4_ (9 and 10 mm) showed good activities against *E. coli* and *C. albicans* species, respectively. The only recorded activity against the growth inhibition of *P. aeruginosa* species was recorded for the compound with a 4-ClC_6_H_4_ substituted group (10 mm). The compound with an unsubstituted phenyl ring presented the most potent antifungal activity against *C. albicans* species (14 mm). The MIC values of the different assessments presented the potent values for compounds with Ar = C_6_H_5_, 4-CH_3_C_6_H_4_, 4-FC_6_H_4_ against *B. subtilis* species, along with 4-ClC_6_H_4_ against *S. aureus* species with MIC values at 2 μg mL^−1^.^83^

**Scheme 8:**
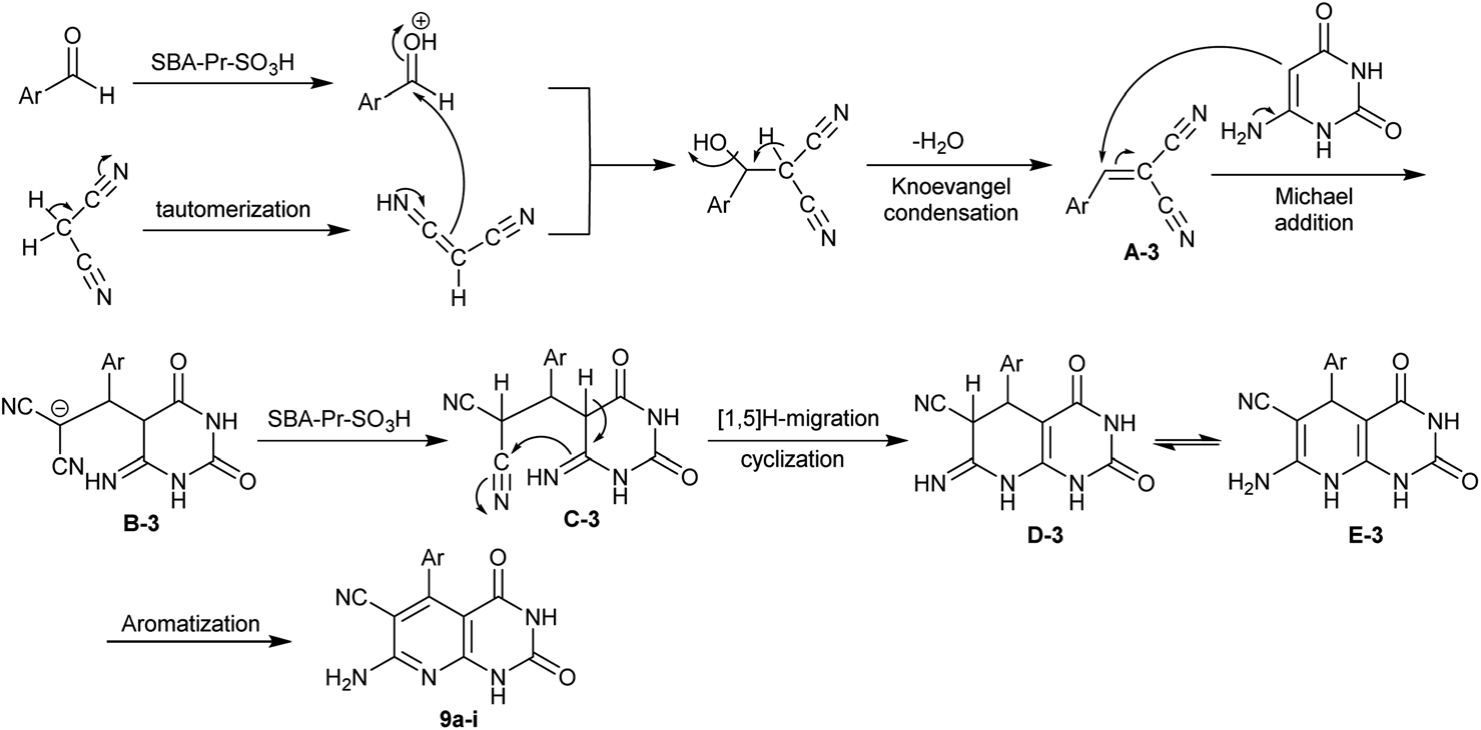
The plausible mechanism for the synthesis of the target pyridopyrimidines.

In a sequence of the mechanistic routes for the interaction of the reacted substrates, two basic steps involved the Knoevenagel condensation and Michael addition. The nano-catalyst enables the activation of the groups for both steps. The systematic sequence involved the activation of the aldehydes, tautomerization of the nitriles, Knoevenagel condensation, Michael addition with amino-uracil, cyclization, and aromatization (Scheme 8).^83^

**Scheme 9:**
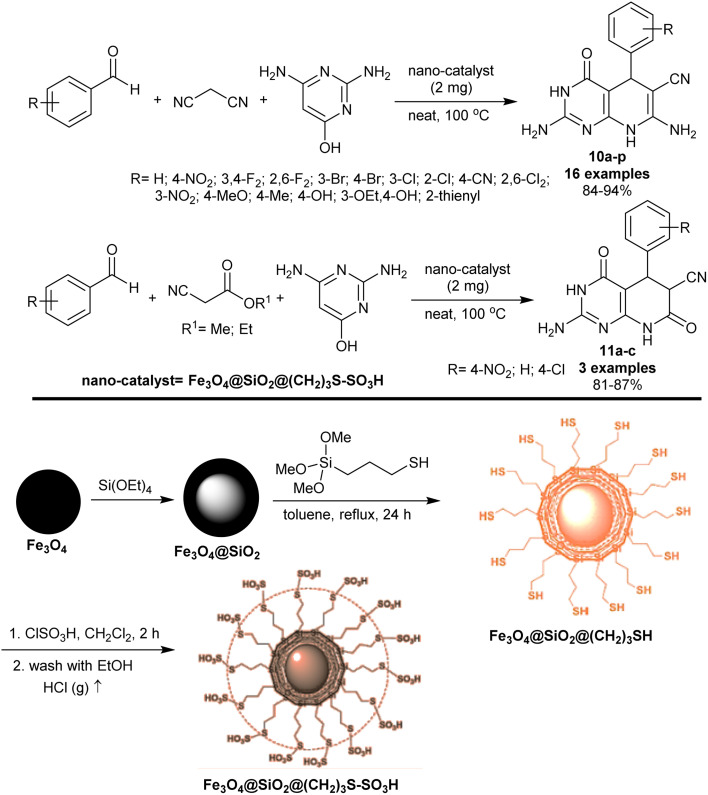
Synthesis of tetrahydro/and hexahydro-pyridopyrimidines.

In 2018, Moradi *et al.*^86^ developed the synthesis of tetrahydropyrido[2,3-*d*]pyrimidines 10a–p and hexahydropyrido[2,3-*d*]pyrimidines 11a–c using Fe_3_O_4_@SiO_2_@(CH_2_)_3_S–SO_3_H nano-magnetic catalyst (Scheme 9). Particularly, a multicomponent one-pot procedure was used to prepare the target compounds under heating and catalytic conditions. Consequently, reactions of aryl aldehydes with 2,6-diaminopyrimidin-4-ol and active methylene components, for instance, malononitrile or methyl 2-cyanoacetate or ethyl 2-cyanoacetate yielded compounds 10 and 11, respectively. The substitution with *para*-alkyl or hydroxy groups on the phenyl ring of the aldehyde or the use of thiophene-2-carbaldehyde tended to reduce the product yield. The magnetic nanocatalyst was prepared from Fe_3_O_4_ magnetic nanoparticles with sonicated silica-coated magnetic nanoparticles in anhydrous toluene. Successively, 3-(trimethoxysilyl)-1-propanethiol was added to the suspension and the mixture was refluxed with continuous stirring to give Fe_3_O_4_@SiO_2_@(CH_2_)_3_SH. At the end, chlorosulfonic acid was dropwise added to the mixture at 25 °C to produce the desired Fe_3_O_4_@SiO_2_@(CH_2_)_3_S–SO_3_H nano-catalyst.

**Scheme 10:**
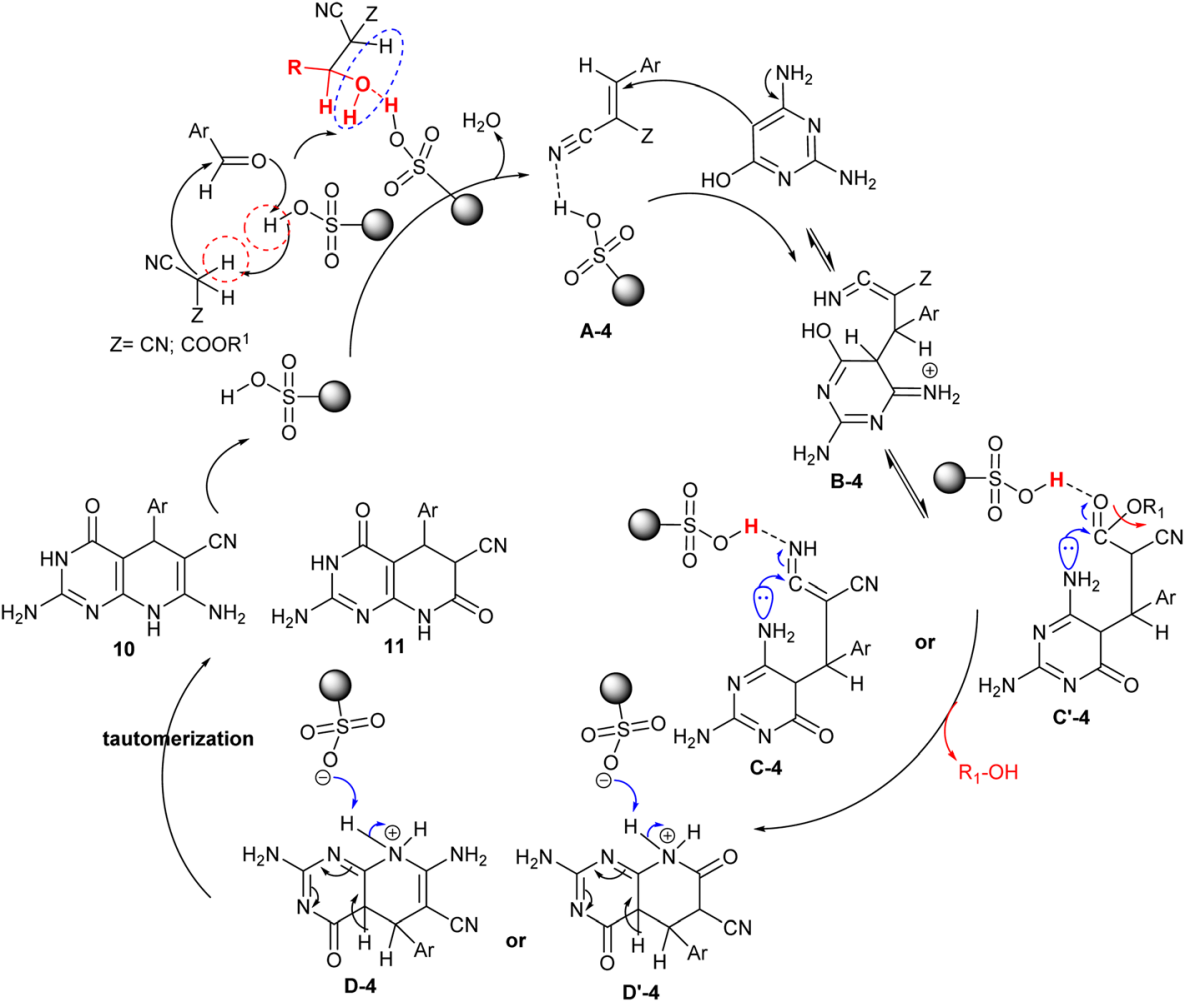
The plausible mechanism for the synthesis of pyridopyrimidines.

The proposed mechanism for the cyclization of the target products, pyridopyrimidines 10 and 11 is shown in Scheme 10. In particular, the acidic character of the sulfonic group of the nanocatalyst increased the reaction rate in the Knoevenagel condensation, Michael addition, and tautomerization steps. Thus, the condensation of the aryl aldehydes with alkyl cyanoacetates generated the arylidene intermediates A-4, which interacted with 2,6-diaminopyrimidin-4-ol through Michael addition sequence to form the intermediates B-4. Tautomerization of intermediates B-4 and subsequent intramolecular cyclization of the intermediates C-4 and C′-4 generated the cyclized intermediates D-4 and D′-4. The SO_3_ group of the nanocatalyst in this mechanism donates a proton for the neutralization of the imino group or participates in the abstraction of the alkoxy group by the release of alcohol from the second possible formed intermediate C′-4. The second step of the nano catalyst is to participate in the abstraction of a proton from intermediates D-4 and D′-4 to neutralize the positive charged that formed on the nitrogen atom after the cyclization of the pyridine ring. Tautomerization of intermediates D-4 and D′-4 gave the anticipated bicyclic products 10 and 11 with the nano-catalyst release and recyclable.^86^

**Scheme 11:**
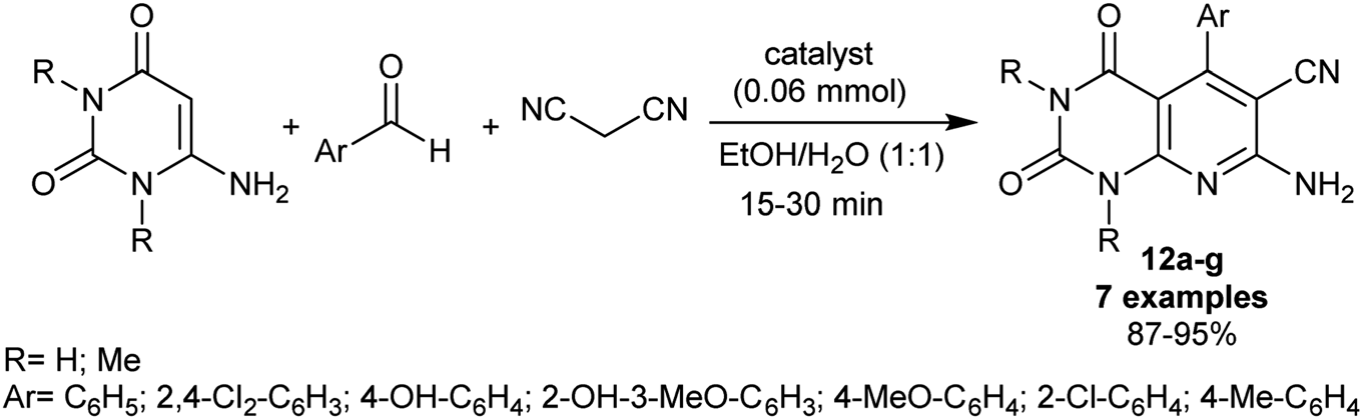
Nano-catalytic, and green synthesis of pyrido[2,3-*d*]pyrimidines.

In 2019, Farahmand *et al.*^87^ performed the synthesis of pyrido[2,3-*d*]pyrimidine analogs by the use of an efficient Mn-ZIF-8@ZnTiO_3_ nano-catalytic nanocomposite. The produced enaminonitriles 12a–g are considered reactive synthons in the preparation of future privileged heterocycles of promising biological potency. Thus, the synthesis of these compounds 12a–g was accomplished in 87–95% yields through one-pot three-component reactions of 6-aminouracil or 1,3-dimethyluracil with aryl aldehydes, and malononitrile in ethanol/water mixture under gently heating, and nano-catalytic conditions (Scheme 11). Some of the heterocycles in this series were beforehand synthesized under diverse catalytic conditions as mentioned above.^82,83^ The nanocomposite was prepared by a sol–gel technique comprising the treatment of manganese(ii) nitrate tetrahydrate with 2-methylimidazole (1 : 2 molar ratio) in methanol under heating conditions to give Mn-ZIF-8. Next, ZnTiO_3_ was prepared by treatment of ethanol solutions of citric acid with tetraethyl *ortho*-titanate, and zinc acetate under heating conditions. A mixture of zinc titanate and Mn-ZIF-8 (2 : 1) was refluxed in ethanol to provide the desired Mn-ZIF-8@ZnTiO_3_ nano-catalyst. The smart features of the nano-catalyst recyclability, green solvents, increased reaction rate, low costs, reverent yields, and simplicity of workup are attractive advantages of this procedure along with the ease of nanocatalyst preparation.

**Scheme 12:**
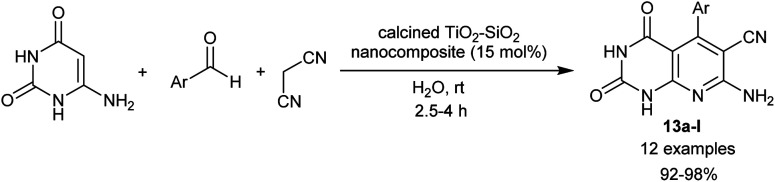
Three-component synthesis of bicyclic tetrahydropyrido[2,3-*d*]pyrimidines.

In the preceding procedures, Abdolmohammadi and Balalaie^84^ employed the use of zirconium dioxide nanoparticles as a useful heterogeneous nano-catalyst in a green procedure for the synthesis of tetrahydropyrido[2,3-*d*]pyrimidines integrated enaminonitrile motif in 86–97% yields through three-component one-pot reactions of aryl aldehydes, malononitrile, and amino-uracil. More recently, the nano-catalytic conditions were applied using calcined TiO_2_–SiO_2_ nanocomposite for the effectual synthesis of tetrahydropyrido[2,3-*d*]pyrimidines 13a–l incorporated enaminonitrile moiety (Scheme 12). Thus, Yaltaghian-Khiabani *et al.*^88^ have estimated this procedure to utilize the benefits of the nano-catalyst as it revealed distinctive features, for instance, non-toxic, inexpensive, moisture stable, and recyclable. In this procedure, three-component one-pot reactions of amino-uracil, aryl aldehydes, and malononitrile in water at ambient temperature under the nanocatalytic conditions produced the desired bicyclic pyridopyrimidine products. The green protocol herein was employed to obtain superior yields of products, high reaction rates, simple operation, and mild conditions of the reactions.

**Scheme 13:**
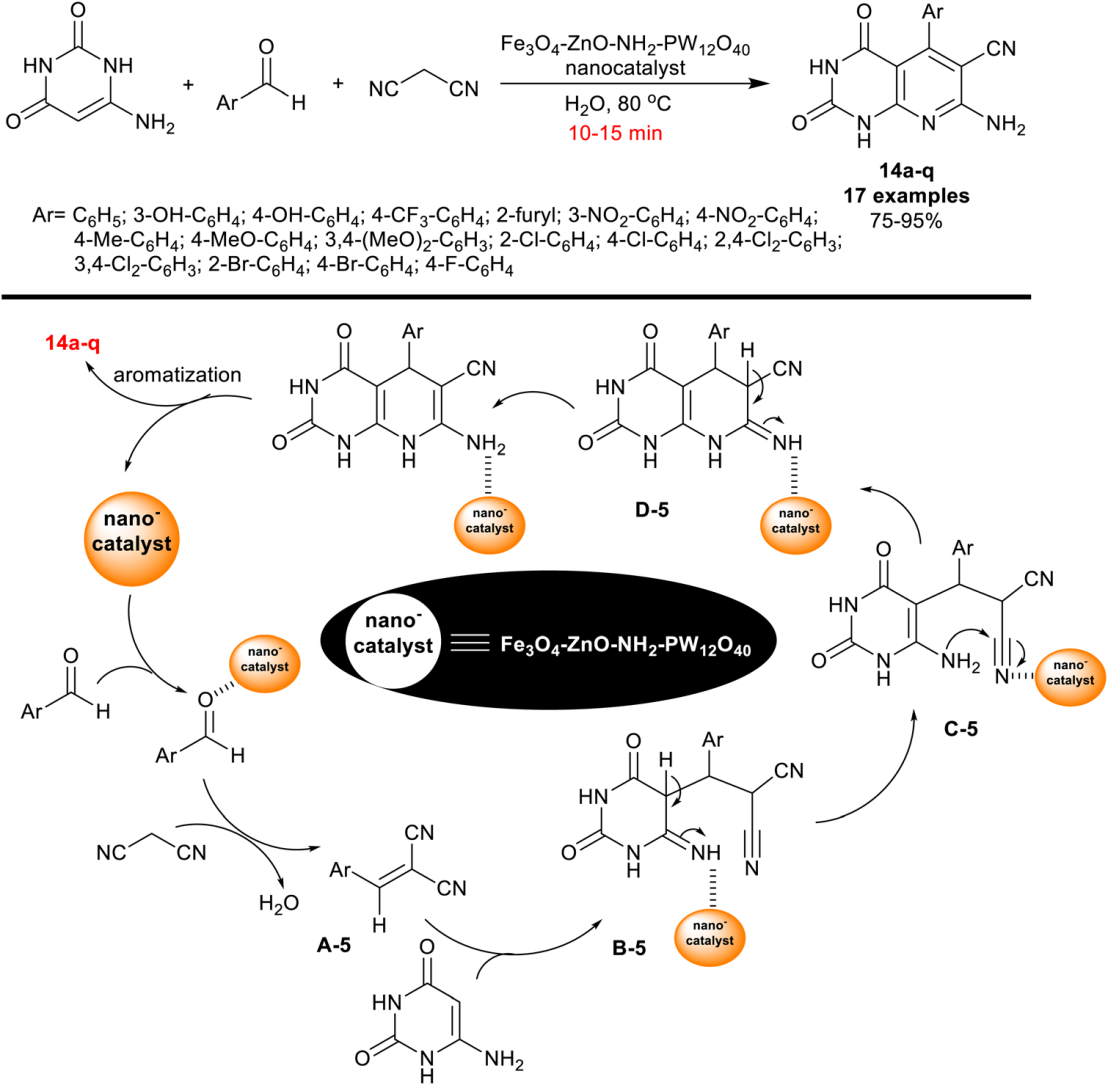
Nanocatalyzed synthesis of enaminonitriles of tetrahydro-pyridopyrimidines.

Also, in 2019, Farokhian *et al.*^89^ utilized Fe_3_O_4_-ZnO-NH_2_-PW_12_O_40_ nanocatalyst for the green synthesis of tetrahydropyrido[2,3-*d*]pyrimidines 14a–q. Thus, three-component one-pot reactions of 6-amino-uracil with aryl aldehydes, and malononitrile in water under smooth heating yielded the respective bicyclic products 14a–q (Scheme 13). The applied nanomagnetic catalyst, Fe_3_O_4_·PMO1 (20 mol%) provided reduced reaction time with increasing the reaction rate than the preceding mentioned approach involved the use of nano-MgO (25 mol%), although the same product yield was obtained from compound 14 (Ar = C_6_H_5_).^82^

The hydroxyl substituents on the phenyl ring of the aldehyde along with the heteroaryl aldehydes such as furan-2-carbaldehyde produced decreased product yields (75–81%). As shown from the probable mechanism for the construction of the bicyclic products (Scheme 13), arylidene intermediate A-5 was formed by Knoevenagel condensation between the aldehydes, and malononitrile. The interaction of 6-amino-uracil with the produced alrylidene intermediates A-5 has proceeded through the Michael addition step to generate intermediates B-5. Next, tautomerization, intramolecular cyclization, and aromatization are the final step sequences for the pyridine ring closure to deliver the final products with the nano-catalyst release. The nano-catalyst was reused for ten succeeding runs with retained catalytic activity. The preparation steps of the nanocatalyst involved the preparation of Fe_3_O_4_ nanoparticles by treatment of FeCl_3_·6H_2_O and FeCl_2_·4H_2_O solutions in distilled water under heating and ultrasound conditions. Polyvinyl pyrrolidone was next added gradually. To a sonicated suspension of Fe_3_O_4_ nanoparticles in ethanol, zinc acetate dihydrate was slowly added to give the desired Fe_3_O_4_–ZnO. Subsequently, 3-aminopropyl-trimethoxysilane was added to a sonicated suspension of Fe_3_O_4_–ZnO in dry ethanol to give Fe_3_O_4_–ZnO–NH_2_. Therefore, H_3_PW_12_O_40_ was added to a suspension of Fe_3_O_4_–ZnO–NH_2_ in distilled water to give the investigated nanocatalyst.^89^

**Scheme 14:**
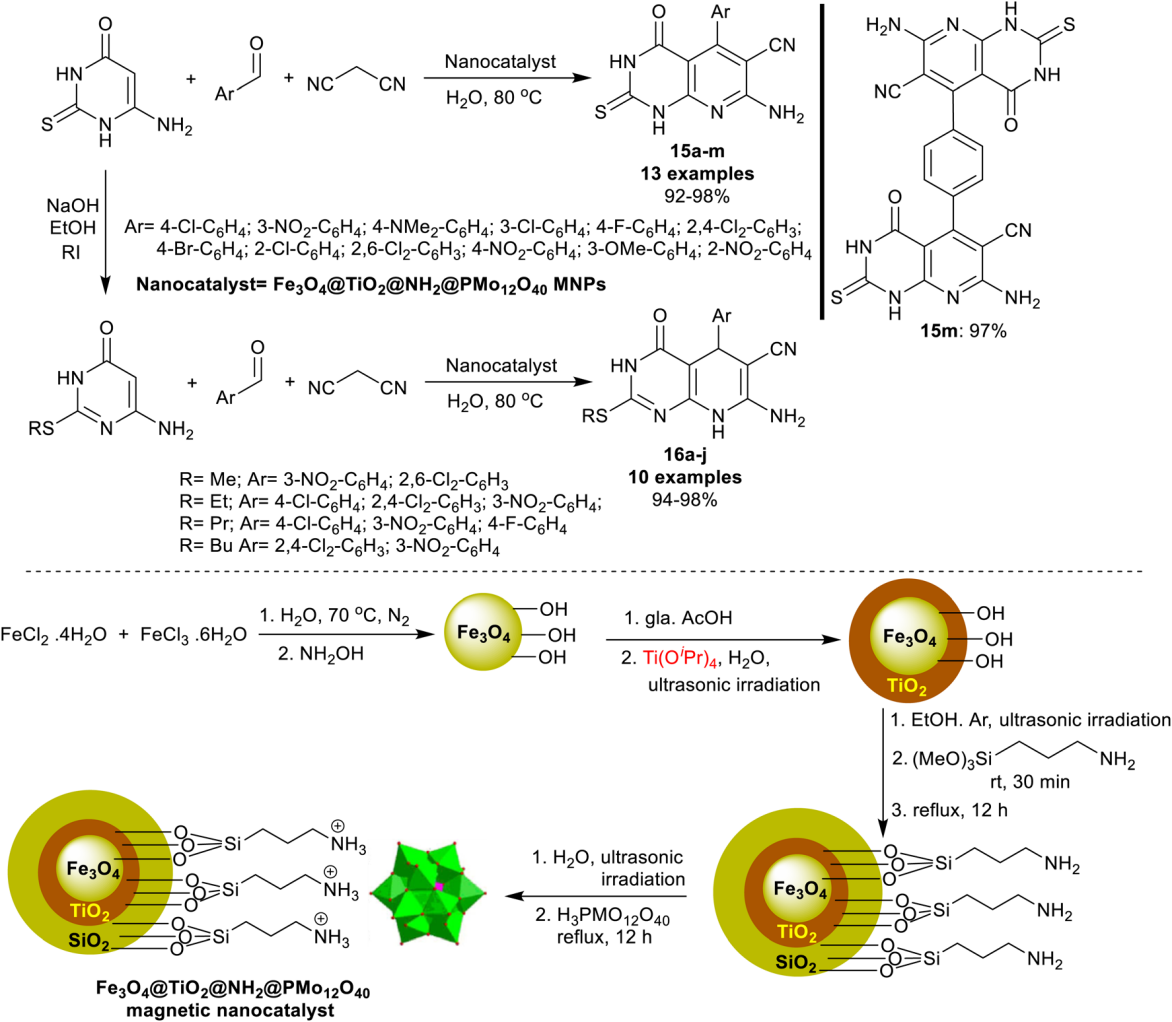
Nanocatalyst preparation, and the green multicomponent synthesis of pyrido[2,3-*d*]pyrimidines.

An efficient procedure was advanced by Saberikhah *et al.*^90^ for the green synthesis of pyrido[2,3-*d*]pyrimidines 15a–m and 16a–j (Scheme 14). Accordingly, one-pot multicomponent reactions of aryl aldehydes activated nitriles, and amino-thiouracil were performed under nano catalytic conditions using magnetic Fe_3_O_4_@TiO_2_@NH_2_@PMo_12_O_40_ to give the bicyclic products 15a–m. Instead, the reactions of 6-amino-2-(alkylthio)pyrimidin-4(3*H*)-one with aryl aldehydes, and malononitrile under the same optimized conditions were used for the preparation of pyridopyrimidines 16a–j. Excellent yields of this procedure indicated the developed nanocatalyst efficiency for the activation of the substrates in this reaction sequence. The nanocatalyst was reusable for eight runs and was prepared by heating iron chloride salts in water, the reaction with hydroxyl amine, and then tetraisopropyl *ortho*-titanate in glacial acetic acid to produce titanium-coated nanoparticles. Treatment of Fe_3_O_4_@TiO_2_ MNPs in ethanol under US conditions and then treatment with (3-aminopropyl)trimethoxysilane gave the desired Fe_3_O_4_@TiO_2_@NH_2_ MNPs. Treatment of the formed nanoparticles with phosphomolybdic acid gave the desired Fe_3_O_4_@TiO_2_@NH_2_@PMo_12_O_40_ nanoparticles.

**Scheme 15:**
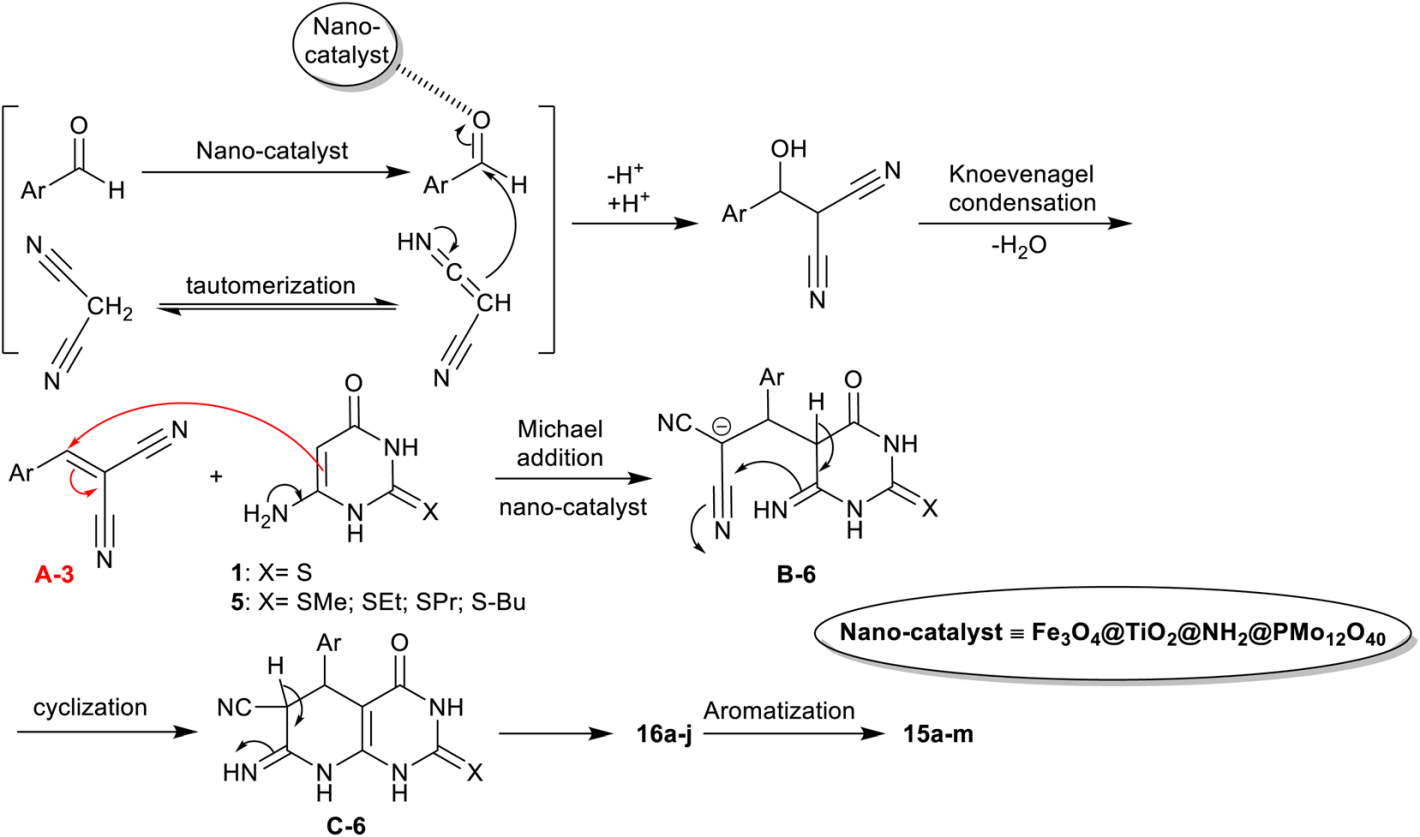
The mechanistic route proposed for the synthesis of pyrido[2,3-*d*]pyrimidines.

The proposed mechanism, as shown in Scheme 15,^90^ indicated the role of the nanocatalyst. Thus, the nanocatalyst activated the aldehyde carbonyl group for the Knoevenagel condensation with the active methylene group of the nitrile, and hence enable the tautomerization of the amino-uracil in the Michael addition step. The Knoevenagel condensation led to the formation of the intermediate A-6, which followed the Michael addition reaction with amino-uracil to shape the intermediate B-6. The cyclization of the intermediate B-6 followed by [1,3]H migration yielded the products 16, and aromatization produced the products 15.

**Scheme 16:**
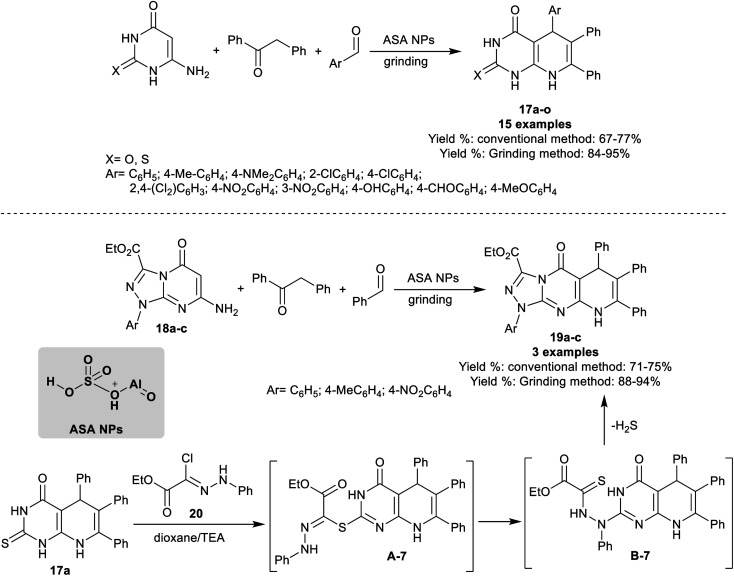
Synthetic route for the preparation of dioxo/thioxo-dihydropyrido[2,3-*d*]pyrimidines.

Abdelrazek *et al.*^91^ have progressed the synthesis of dioxo/thioxo-dihydropyrido[2,3-*d*]pyrimidines 17a–o and tetrahydropyrido[1,2,4]triazolopyrimidines 19a–c employing three-component one-pot reactions under catalytic conditions using aluminate sulfonic acid nanoparticles. The products were prepared using conventional grinding procedures. The best yields were achieved by the green grinding method (84–95%). The best yield (77%) was achieved by applying the reaction of 6-amino-2-thioxo-2,3-dihydropyrimidin-4(1*H*)-one with 1,2-diphenylethan-1-one and 2,4-dichlorobenzaldehyde using the conventional method. Specifically, the best yield obtained by the grinding method was recorded for the aryl aldehydes substituted with electron-withdrawing groups, for instance, a chlorine atom, and nitro group. On the other hand, one-pot three-component reactions of ethyl 1-aryl-1,5-dihydro[1,2,4]triazolopyrimidine-carboxylates 18a–c with benzaldehyde, and 1,2-diphenylethan-1-one under the same optimized nano-catalytic conditions gave the desired pyrido[1,2,4]triazolopyrimidines 19a–c (Scheme 16).

In an alternative route, pyrido[2,3-*d*]pyrimidinone 17a reacted with ethyl (*Z*)-2-chloro-2-(2-phenylhydrazono)acetate 20 to afford the desired tricyclic product 19a (Scheme 16). The reaction proceeded through *S*-alkylation, rearrangement, and the release of H_2_S molecules. All the reactions were accomplished under nanocatalytic and solvent-free conditions. The acid character of the nano-catalyst enables the tautomerization of the carbonyl group of 1,2-diphenylethan-1-one, and activated the aldehyde carbonyl group for the condensation stage. The nano-catalyst role was extended for the next steps involving the interactions with the amines through Michael addition steps, and cyclocondensation step with the formation of the products and release of the nano-catalyst.^91^

**Scheme 17:**
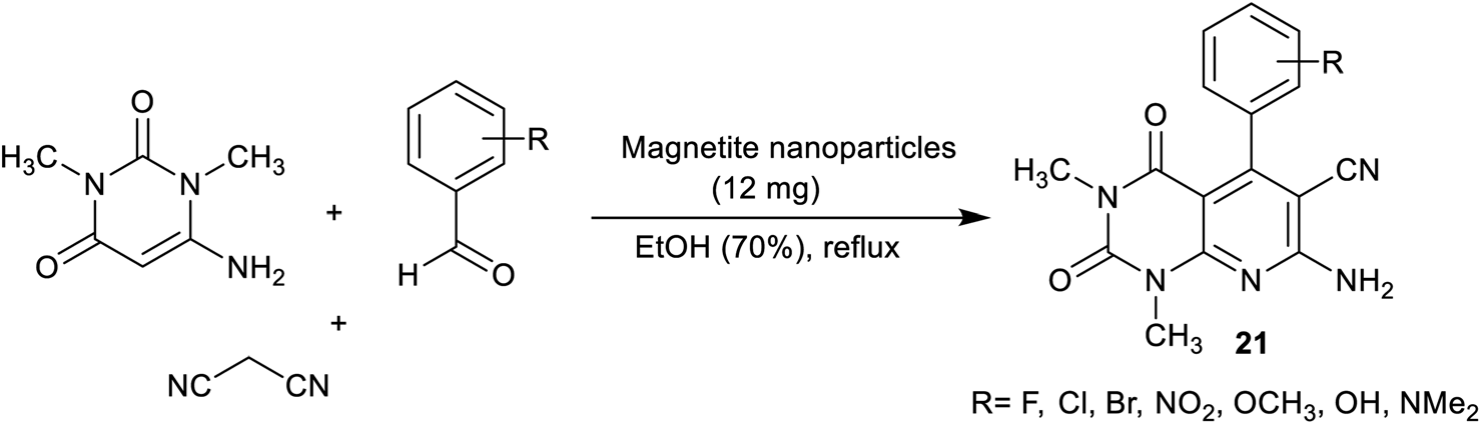
Synthesis of pyrido[2,3-*d*]pyrimidines using magnetic nanoparticles.

More recently, Esmaili *et al.*^92^ have synthesized a series of pyrido[2,3-*d*]pyrimidines 21 using a nano-magnetite complex. In this route, nano-[Fe_3_O_4_@SiO_2_/*N*-propyl-1-(thiophen-2-yl)ethanimine][ZnCl_2_] was applied as an efficient nano-magnetite catalyst. Three-component, and one-pot reactions of amino-uracil with aryl aldehydes and malononitrile in ethanol under heating, and catalytic conditions gave the desired bicyclic pyridopyrimidine products. The method delivered a green procedure, catalyst reusability, amplified the reaction rate, and ease of product workup (Scheme 17).

#### 2.2.2 Reactions involved cyclic ketones

**Scheme 18:**
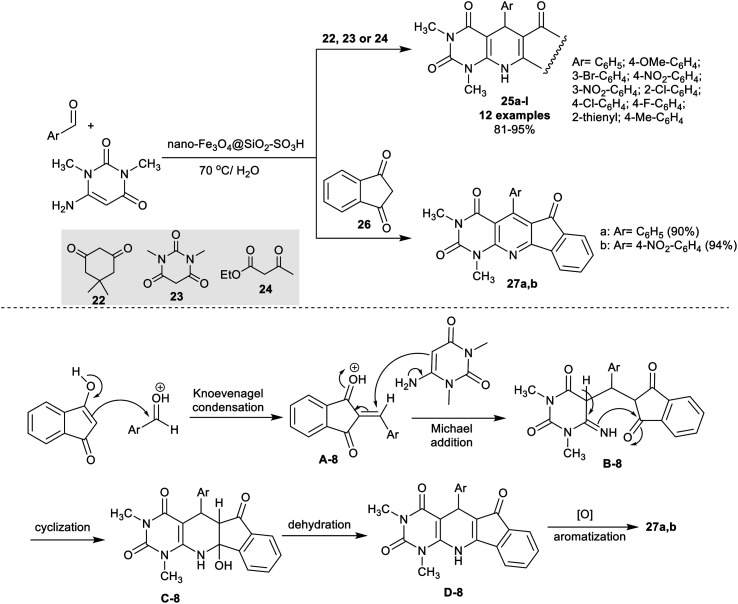
One-pot synthesis of polycyclic pyrido[2,3-*d*]pyrimidines under green, and nano-catalytic conditions.

The method of Nemati and Saeedirad^5^ in 2013 concerned the utility of nano-Fe_3_O_4_@SiO_2_–SO_3_H for the effectual one-pot three-component synthesis of the polycyclic system-incorporated pyrido[2,3-*d*]pyrimidine motif. The nano-catalyst is recyclable for up to three runs, and the procedure was accomplished in water “green solvent” under gentle heating conditions. The reactions of aryl aldehydes with amino-pyrimidine-dione, and active methylene compounds, for instance, cyclohexane-dione, or 1,3-dimethyl-pyrimidine-trione or ethyl 3-oxobutanoate 22–24 gave the target products 25a–l in superior yields (81–95%). Consequently, the incorporation of 1*H*-indene-1,3(2*H*)-dione 26 in the reaction system as an active methylene component yielded the tetracyclic systems 27a,b. The products' yield is influenced by the nature of the structured phenyl substituents as the nitro group presented a slightly improved yield in the unsubstituted phenyl ring of the aldehyde. For compounds 25a–l, the yield of the products was noticed higher than 90% for most of the products, and reduced yields were obtained from the *ortho*-chloro substituents, heteroaryl aldehydes, and electron donating groups at the para position of the phenyl ring. The acid characteristics of the nano-catalyst supported the improved yields and increased reaction rate. The sulfonic group simplifies Knoevenagel condensation, the Michael addition step, and the intramolecular cyclization of the pyridine ring. The last two steps in the conceivable mechanism of this reaction sequence are the dehydration, and oxidative aromatization of the pyridine ring to give products 27a,b (Scheme 18). The oxidative aromatization step was not involved in the mechanistic sequence of products 25a–l.

**Scheme 19:**
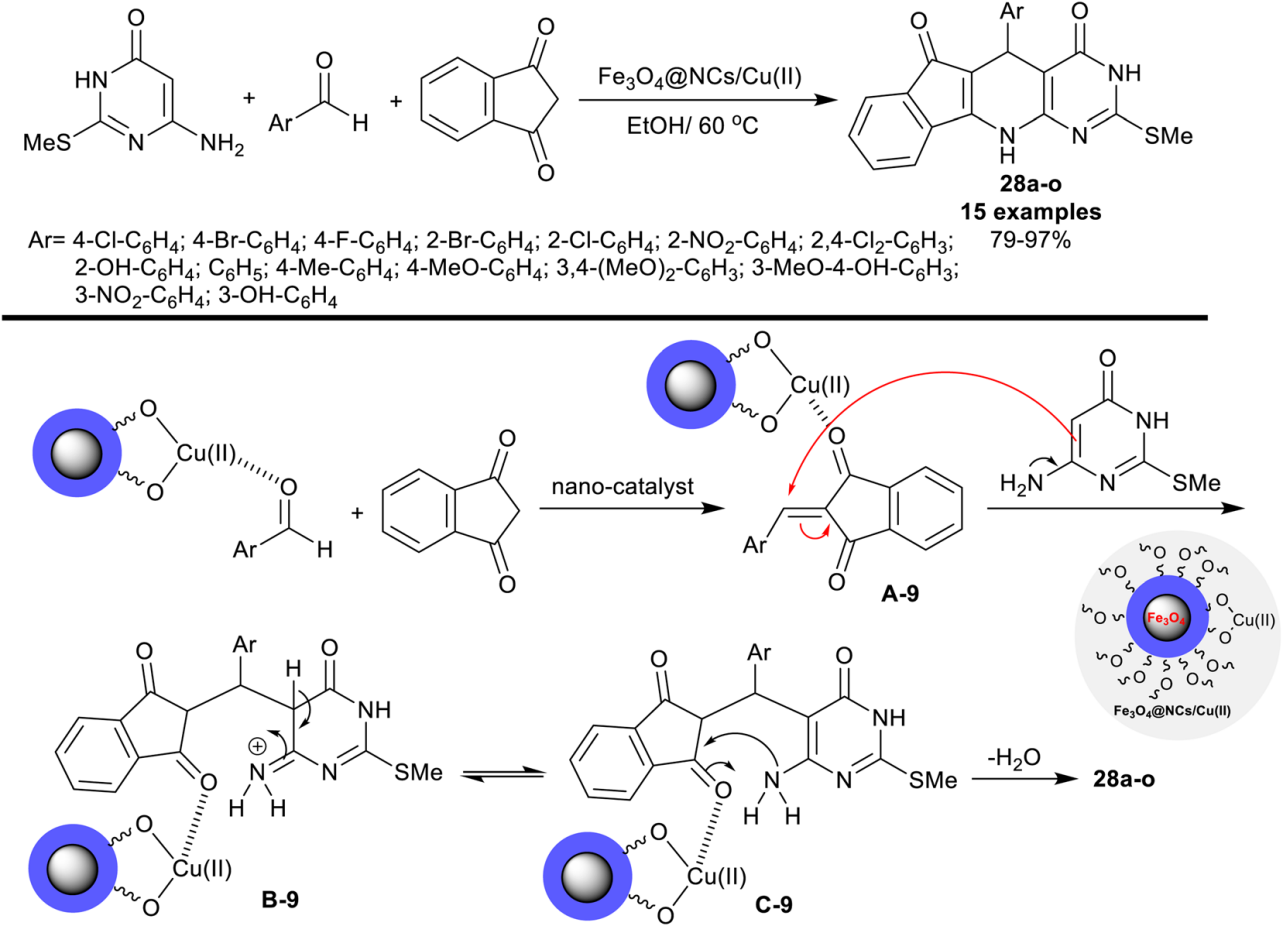
Synthesis and the plausible mechanistic route of indenopyridopyrimidines.

In 2021, Safajoo *et al.*^93^ prepared a series of tetracyclic systems 28a–o using the biosynthesized Fe_3_O_4_@NCs/Cu(ii) magnetic nano-catalyst. The nano-catalyst was prepared by treatment of copper(ii) chloride with Fe_3_O_4_@NCs in a basic medium. In this performance, dihydro-3*H*-indenopyrido[2,3-*d*]pyrimidine analogs were efficiently synthesized by one-pot three-component reactions of 6-amino-2-(methylthio)pyrimidin-4(3*H*)-one with 1*H*-indene-1,3(2*H*)-dione, and aryl aldehydes. The reactions were run in ethanol containing the nano-catalyst under reflux conditions. The conceivable mechanism of this reaction-type as stated in Scheme 19, did not give the spirocyclic products. Instead, the tetracyclic systems 28a–o were obtained through initial activation of the carbonyl group of the aldehydes by the aid of the nano-catalyst for condensation with 1*H*-indene-1,3(2*H*)-dione to produce the desired arylidene intermediates A-9. The intermediate B-9 was formed through the interaction of the intermediates A-9 with amino-pyrimidinone. The cyclocondensation step led to the pyridine ring cyclization to yield the tetracyclic products. The yields of this reaction sequence varied from 79 to 97% based on the structural nature of the phenyl substituents of the aryl aldehydes. The alkyl substituents tended to give lower yields, while the substitution with halogen and electron-withdrawing groups resulted in excellent yields >90%.

**Scheme 20:**
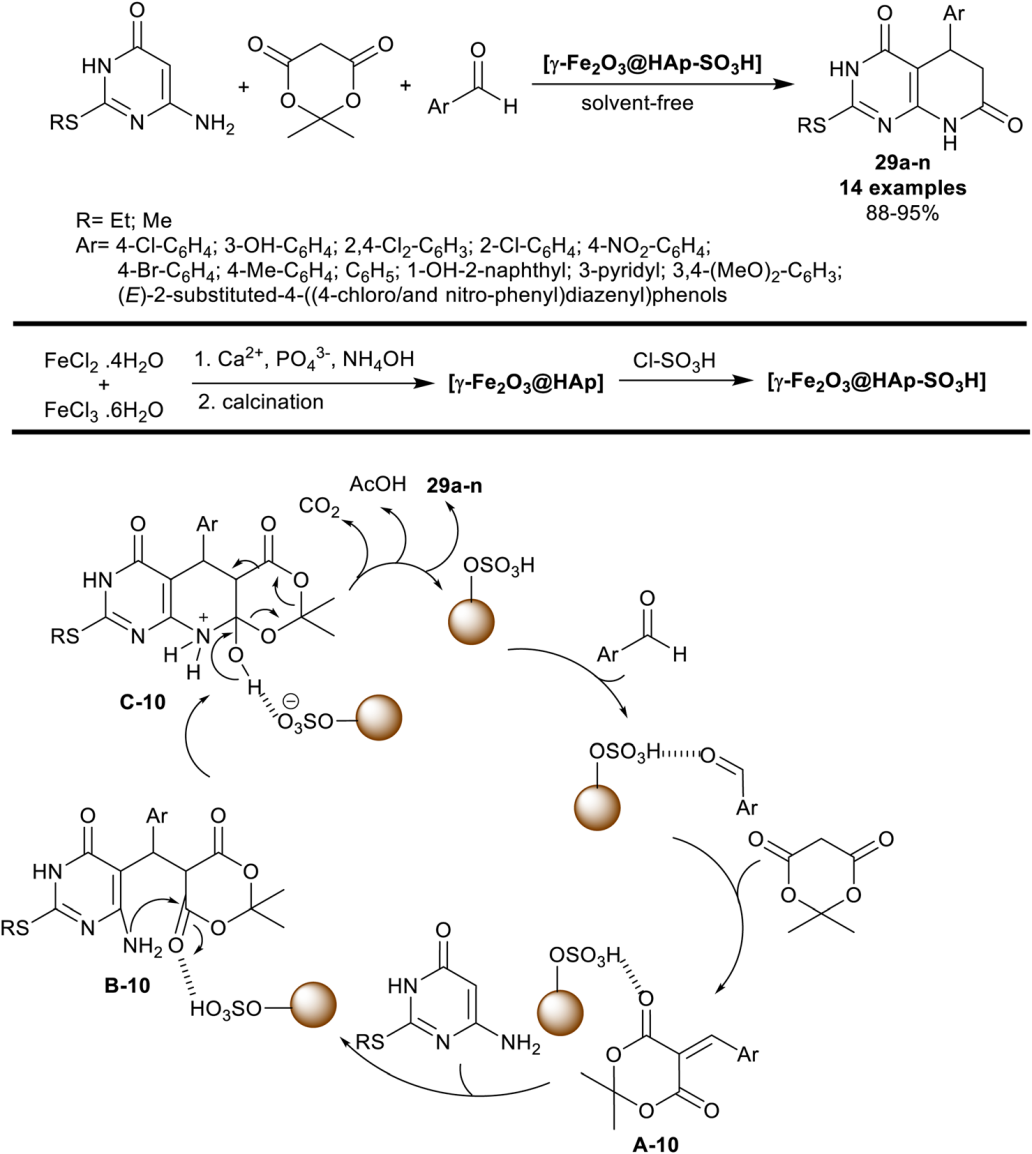
Multicomponent synthesis of dihydropyrido[2,3-*d*]pyrimidine-diones.

Mohsenimehr *et al.*^94^ employed HAp-encapsulated-γ-Fe_2_O_3_ supported sulfonic acid [γ-Fe_2_O_3_@HAp-SO_3_H] nanocatalyst for the synthesis of 5-aryl-2-(alkylthio)-5,8-dihydro-pyrido[2,3-*d*]pyrimidine-diones 29a–n. In this route, the three-component technique was applied in the reactions of 6-amino-2-(alkylthio)pyrimidin-4(3*H*)-one, Meldrum's acid, and aryl aldehydes in a one-pot procedure to give the bicyclic pyridopyrimidines 29a–n (Scheme 20). The reactions were run under gentle heating and solvent-free conditions. The green procedure herein under the nano-catalytic conditions provided tremendous yields of the products (88–95%) with an amplified reaction rate. In all cases, the product yield is influenced by the type of substituents at the phenyl ring of the aryl aldehyde, and the alkyl group linked to the sulfur atom of the amino-pyrimidinone substrate.

The nano-catalyst (Scheme 20) was prepared by the treatment of iron(ii) chloride with iron(iii) chloride solutions, then calcination, and reaction with chlorosulfonic acid. Commonly, the process of the product formation involved the pyridine ring closure through the formation of C–N, two C–C bonds, and cleavage of the 1,3-dioxan-4-one ring. The proposed mechanism involved Knoevenagel condensation/Michael addition cascade courses. The formed arylidene intermediates A-10 interacted with 6-amino-2-(alkylthio)pyrimidin-4(3*H*)-one through a Michael addition step to generate intermediates B-10. The intramolecular nucleophilic attack of the exocyclic amino group at the carbonyl group generated intermediates C-10. Subsequent dioxanone ring cleavage caused the formation of the products 29a–n with the release of carbon dioxide, and acetic acid molecules. The nano-catalyst participated in the activation of all steps to increase the reaction rate with enhanced yields.^94^

**Scheme 21:**
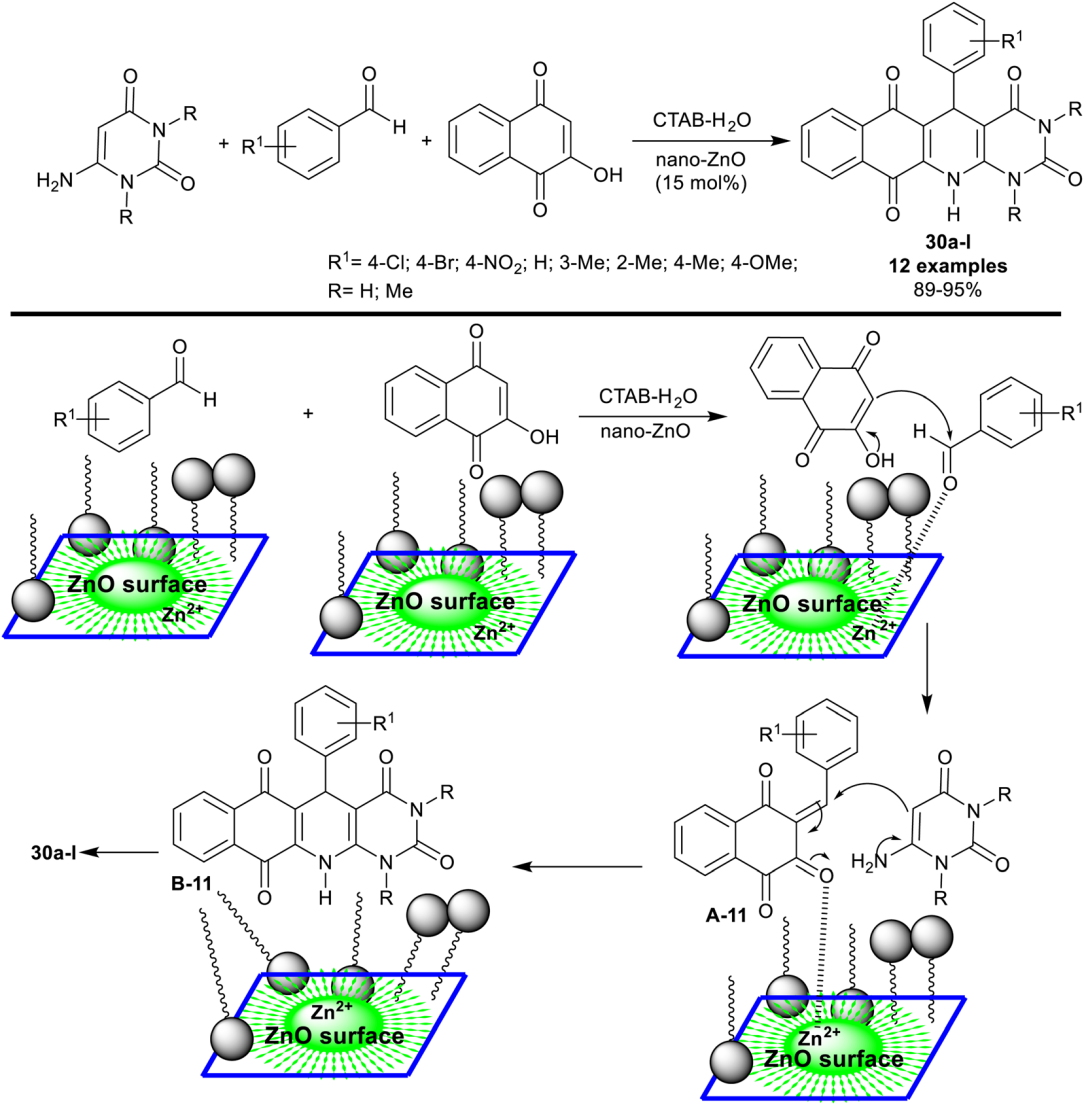
Synthesis of benzopyrimido[4,5-*b*]quinoline-tetraones.

In 2015, Siddiqui *et al.*^95^ reported the synthesis of tetracyclic systems incorporated benzopyrimido[4,5-*b*]quinoline skeleton 30a–l. The three-component synthesis was accomplished through a one-pot procedure under heating and nano-catalytic conditions of zinc oxide. Therefore, reactions of 6-aminouracil with aryl aldehydes, and 2-hydroxynaphthalene-1,4-dione in CTAB (80 mM) – water (admicellar system) containing nano-zinc oxide (15 mol%) gave the anticipated products 30a–l (Scheme 21). The nano-catalyst can be efficiently recycled and reused during this green procedure. In this step, ethyl acetate was added to the reaction mixture with continuous stirring until the product of the reaction was dissolved. Consequently, two layers were formed, including the nano-catalyst in the aqueous layer to be reused. This procedure efficiently produced the products in excellent yields of 89–95%. The conceivable mechanism for the synthesis of compounds 30a–l is presented in Scheme 21. The admicellar system provided a hydrophobic area, in which the substrates can be closer to each other over the surface of zinc oxide nanoparticles. The nano-catalyst supported the generation of admicelles in the water aqueous medium and thus increased the reaction rate. Siddiqui *et al.*^96^ have published an alteration of the experimental data of their work regarding compounds 30a–l (*e.g.* IR, ^1^H-NMR, ^13^C-NMR, MS, and elemental analysis).

**Scheme 22:**
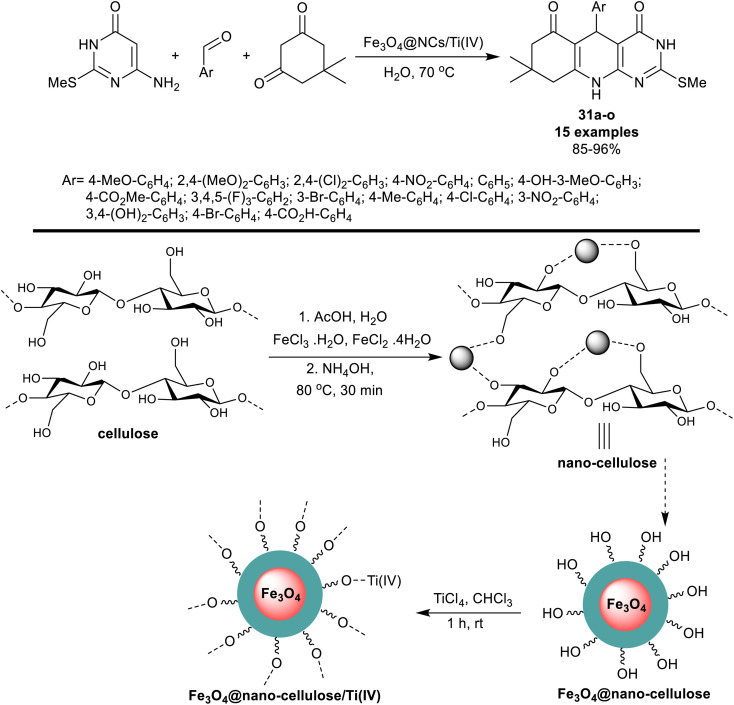
Synthesis of tetrahydropyrimido[4,5-*b*]quinolines.

More recently, Mirjalili *et al.*^97^ investigated the utility of Fe_3_O_4_@nano-cellulose/Ti(iv) as a proficient nanocatalyst for the synthesis of tetrahydropyrimido[4,5-*b*]quinoline analogs 31a–o. Consequently, a one-pot technique was applied in the reactions of 6-amino-2-(methylthio) pyrimidin-4(3*H*)-one with aryl aldehydes, and dimedone under heating conditions in the water. The green methods provided excellent yields of the products and reduced reaction times corresponding to the proficiently of the nanocatalyst to form the desired products. The nano-catalyst was prepared from cellulose by the treatment with metal salt solutions in the presence of acetic acid followed by heating with hydroxyl amine to generate the nano-cellulose supported by the magnetic nano-metal oxide. Further treatment of Fe_3_O_4_@nano-cellulose with titanium(iv) chloride in chloroform yielded the respective Fe_3_O_4_@nano-cellulose/Ti(iv) magnetic nano-catalyst (Scheme 22). The ease of preparation along with the reusability of the nano-catalyst is a beneficial advantage of this method. The cyclization of the pyridine ring was achieved through the formation of C–N, and two C–C bonds including the interaction of the heteroaryl amines with the aldehyde carbonyl group and the active methylene adjacent to the carbonyl group in the dimedone skeleton. Three main steps in this sequence elaborated the Knoevenagel condensation, Michael addition type, and cyclocondensation.

**Scheme 23:**
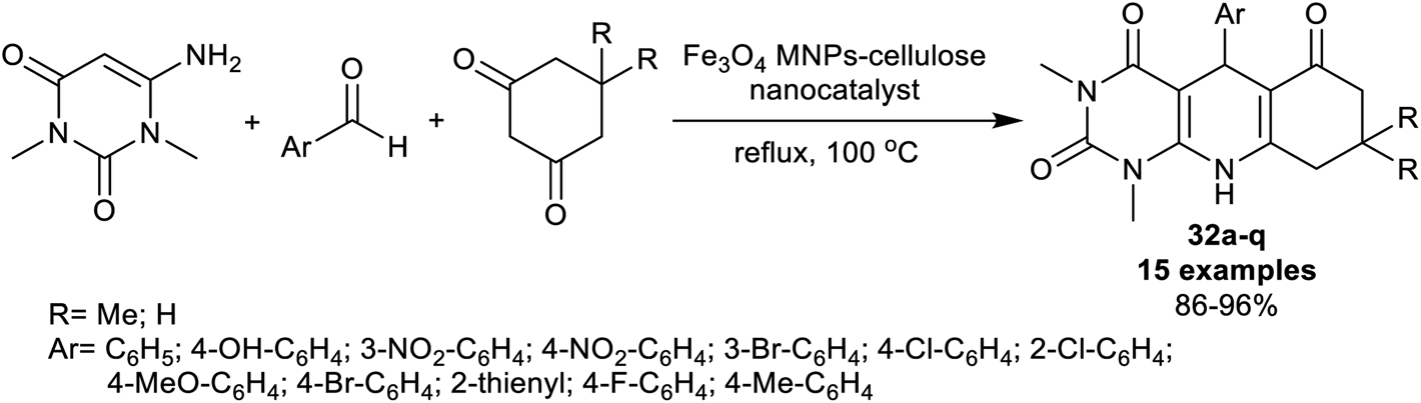
Synthesis of tetrahydropyrimidoquinolines.

On the other side, a series of disubstituted, and tetrasubstituted tetrahydro-pyrimido-quinolines 32a–q were synthesized by Edjlali *et al.*^98^ under nano-catalytic conditions. Thus, three-component one-pot reactions of *N*,*N*-dimethyl-aminouracil with aryl aldehydes, and cyclohexandiones by heating in the attendance of Fe_3_O_4_ nano-catalyst supported on cellulose gave the desired products 32a–q (Scheme 23). The procedure is a green protocol since the reactions were run in water along with the low cost of this technique. Also, this nano-catalyst is recyclable and provided the simple preparation of the nanocatalyst, and improved yields of the products with the ease of product separation and isolation. The extraordinary influence of the substitution of the phenyl ring with a *para*-nitro group on the product yields was noted, whereas the bromine and hydroxy groups decreased the product yields. The nano-catalyst was prepared by treating cellulose in a solution of ferric, and ferrous sulfates and maintaining the pH at 10 by the addition of a solution of ammonium hydroxide (25%).

#### 2.2.3 Reactions involved cyanoacetyl motif

**Scheme 24:**
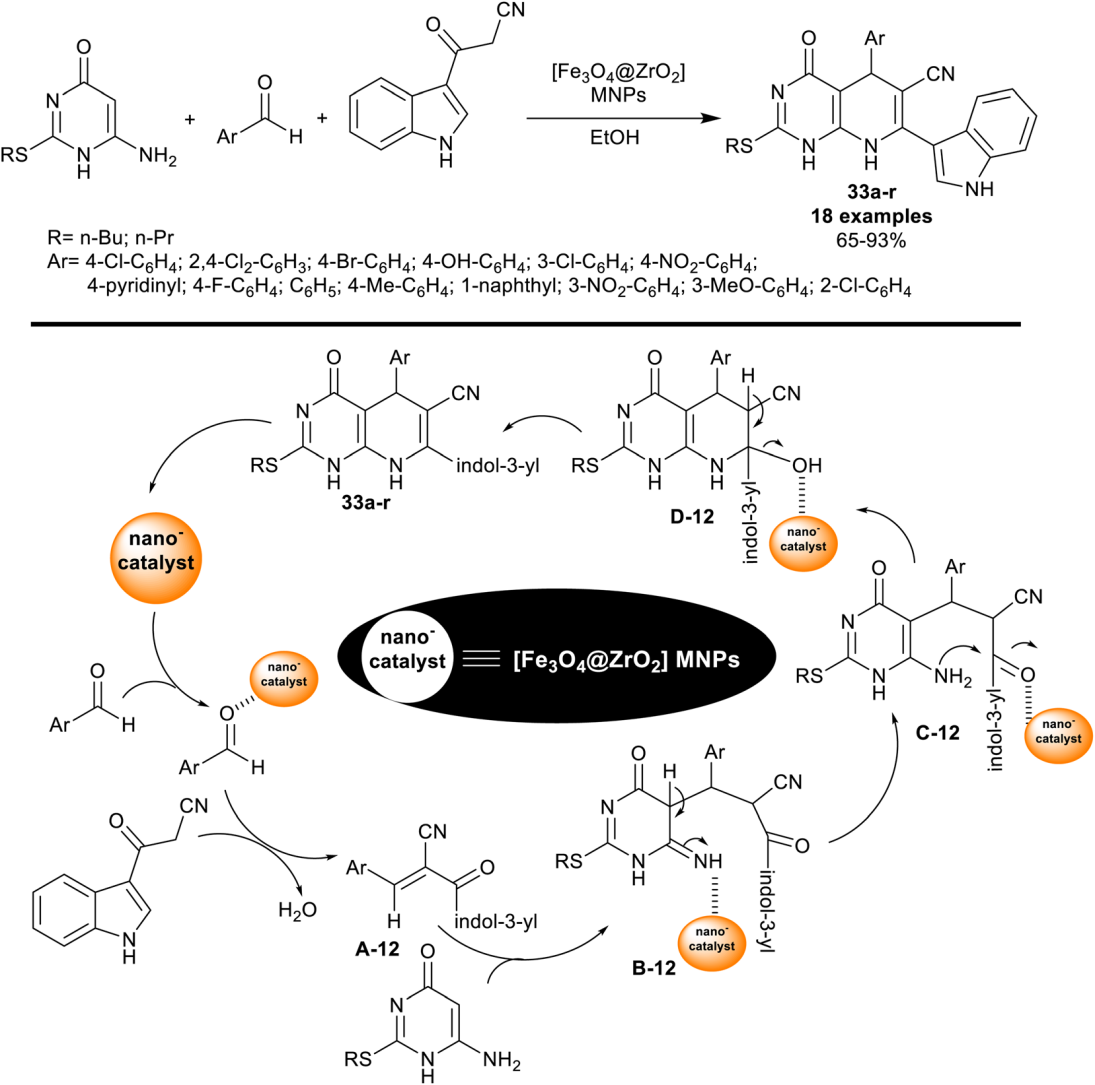
Multicomponent reactions, and the reasonable mechanism for the synthesis of indolyl-pyridopyrimidines.

The indole analogs involve benefited skeleton in pharmaceutical, drugs, and material science fields. Such indole alkaloids and drugs incorporated indole nuclei presented attractive biologically active features.^99–102^ Kheirkhah *et al.*^103^ developed the synthesis of pyrido[2,3-*d*]pyrimidines 33a–r using [Fe_3_O_4_@ZrO_2_] magnetic nanoparticles. In this procedure, thioalkyl-aminouracil reacted with aryl aldehydes, and 3-cyanoacetylindole in a one-pot procedure to provide the required products 33a–r (Scheme 24).

Particularly, the nanocatalyst enhanced the yields of the products (65–93%), reduced the reaction time, and enabled nanocatalyst reusability. Such reactions of this system produced bicyclic pyridopyrimidines linked to the indole motif in a route to enriching the biological significance of this type of molecule. As estimated from the plausible mechanism in Scheme 24, the reactions involved the activation of the aldehyde carbonyl group for Knoevenagel condensation with the active methylene group of 3-cyanoacetylindole to produce the arylidene intermediates A-12. The thioalkyl-aminouracil tended to Michael's addition steps to the formed arylidene intermediates A-12 to generate the intermediates B-12. [1,3]H-migration followed by intramolecular cyclization, and condensation steps gave the products 33a–r. The pyridine ring was cyclized through the formation of C–C “involved the arylidene formation”, and C–N bonds “involved the intramolecular cyclization”.^103^

**Scheme 25:**
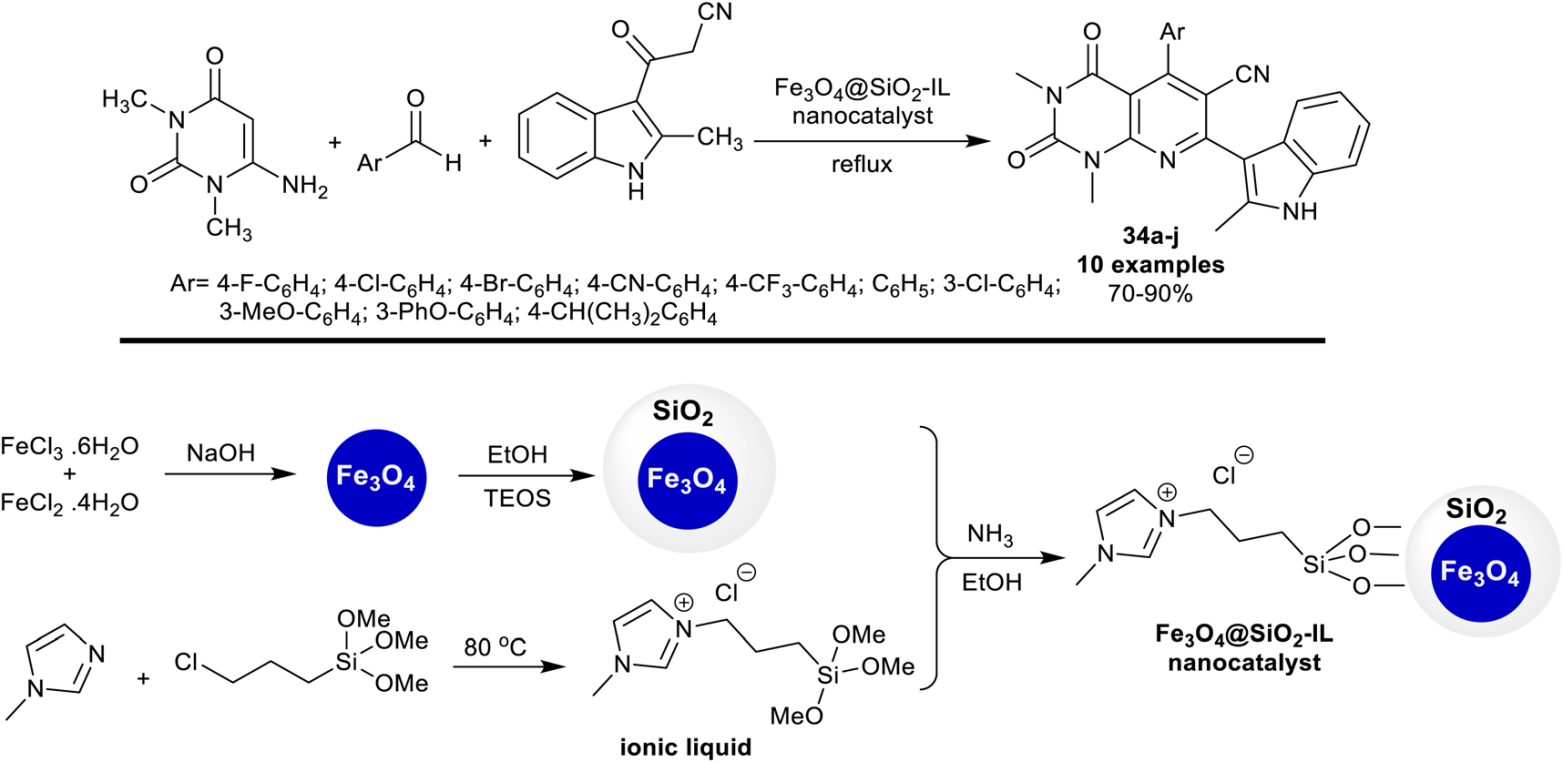
Preparation of the Fe_3_O_4_@SiO_2_-supported ionic liquid nanocatalyst for the synthesis of indolyl pyrido-pyrimidines.

In 2018, Mamaghani *et al.*^104^ utilized Fe_3_O_4_@SiO_2_-supported ionic liquid nanocatalyst for the synthesis of alkyl-indolyl pyrido[2,3-*d*]pyrimidines 34a–j. Thus, one-pot multicomponent reactions of dialkyl amino-uracil with aryl aldehydes and 2-methyl-3-cyanoacetylindole under the optimized conditions gave the anticipated products 34a–j. The nanocatalyst acts as an ionic liquid supported on Fe_3_O_4_@SiO_2_ nanoparticles for the activation of the reactions of the substrates by the interaction with the carbonyl group of the aryl aldehyde, and an amino group of *N*,*N*-dialkyl amino-uracil, and was released from the reaction to be reused. The final step of this proposed mechanism involved the oxidation of the 7-(methyl-1*H*-indolyl)-hexahydropyridopyrimidine intermediates to form the aromatized pyridine ring. The nanocatalyst was simply prepared by the treatment of ferric chloride, and ferrous chloride solutions under an Ar atmosphere with sodium hydroxide solution (10 M) under continuous stirring and ultrasonic irradiation conditions. The ionic liquid solution was prepared by refluxing 1-methylimidazole with (3-chloropropyl)trimethoxysilane under an Ar atmosphere. Treatment of the prepared magnetite nanoparticles in ethanol with the ionic liquid solution under stirring and ultrasound conditions gave the desired nanocatalyst (Scheme 25).

Compounds 34a–j (Scheme 25) were assessed as antibacterial agents against diverse bacterial species using tetracycline and ciprofloxacin as standard antibiotics. The compounds revealed promising activities, for instance, compound 34g (Ar = 3-Cl-C_6_H_4_) showed supreme effectiveness against *S. aureus* species (12 μg per well; 13 mm), which was similar to that of ciprofloxacin. Compound 34e (Ar = 4-CF_3_-C_6_H_4_) demonstrated potent activity against *E. coli* species (13 mm). Also, compound 34b (Ar = 4-Cl-C_6_H_4_) was the most potent antibacterial agent towards *M. luteus* species (20 mm), while the compounds displayed the least activity *versus P. aeruginosa* species (5–9 mm).^104^

**Scheme 26:**
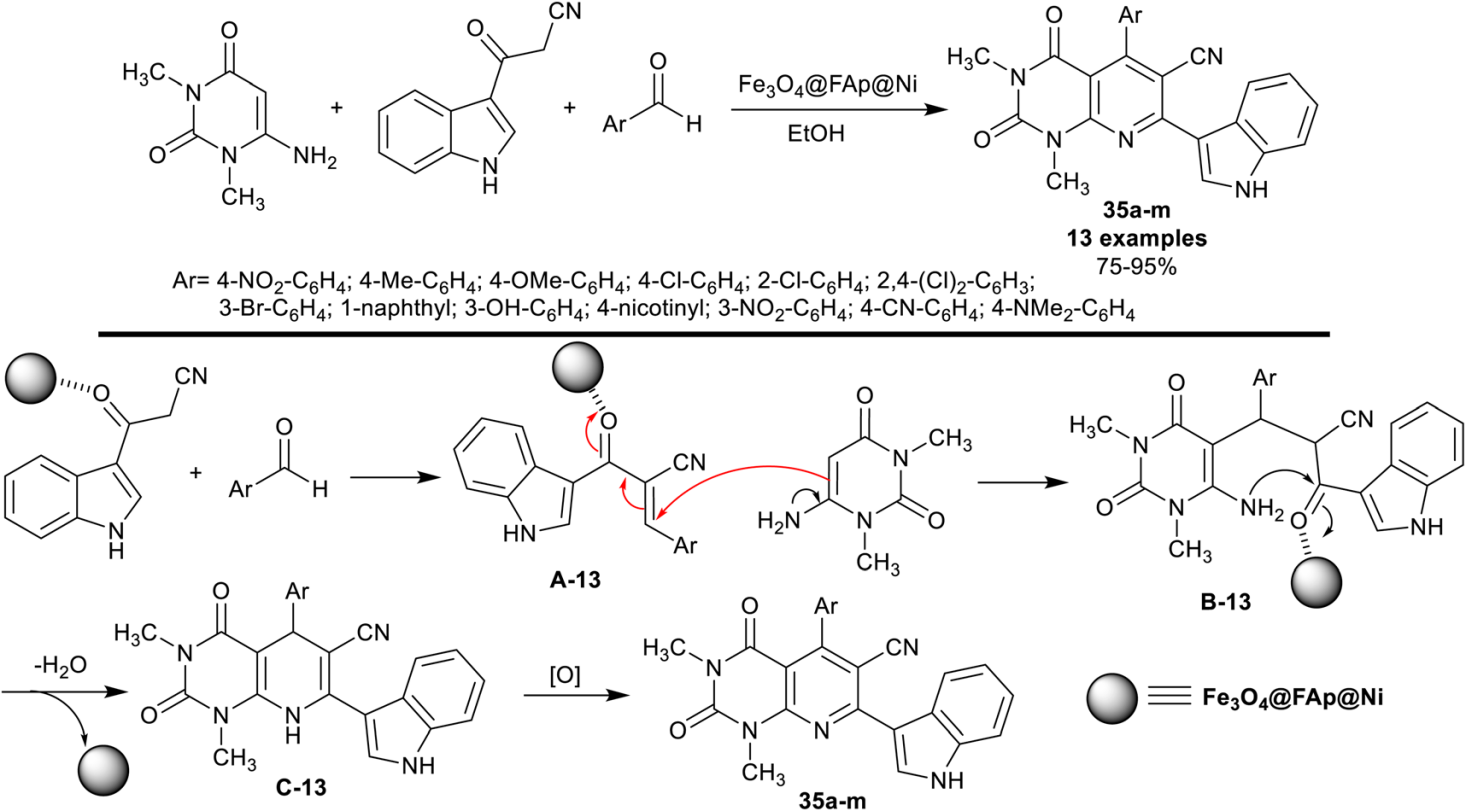
Synthesis and the plausible mechanism of 7-(1*H*-indolyl)-tetrahydropyridopyrimidines.

Dastmard *et al.*^105^ advanced an effectual method for the synthesis of tetrahydro-pyridopyrimidines incorporated in the indole core 35a–m under heating, and nano-catalytic conditions using Fe_3_O_4_@FAp@Ni. Thus, one-pot three-component reactions of 6-amino-1,3-dimethyl-pyrimidine-2,4(1*H*,3*H*)-dione with 3-cyanoacetyl-indole and aromatic aldehydes in ethanol gave the anticipated products 35a–m in good to excellent yields. The utility of the Fe_3_O_4_@FAp@Ni nanocatalyst provided an excellent yield for compound 35a (95%) than applying other catalysts such as Fe_3_O_4_@SiO_2_ nanocatalyst (90%), [γ-Fe_2_O_3_@HAp] (90%), [γ-Fe_2_O_3_@HAp-SO_3_H] (85%), and Ni(NO_3_)_2_·4H_2_O (73%). The series of compounds 35a–d were previously prepared by Naidu *et al.*^106^ under catalytic conditions using InCl_3_. The utility of the Fe_3_O_4_@FAp@Ni nanocatalyst failed to present a suitable procedure for the simple separation of the nanocatalyst, but it provided shortened time for the reactions. The conceivable mechanistic sequence for this type of reaction also involved the formation of Knoevenagel condensation intermediates A-13, generation of Michael-addition intermediates B-13, cyclocondensation, and aromatization of the pyridine ring by the oxidation of the intermediates C-13 (Scheme 26).

The antibacterial activity of compounds 35a–m (Scheme 26) was assessed by the well diffusion technique against *E. coli*, *P. aeruginosa*, *B. subtilis* and *M. luteus* species. A sample solution was evaluated using a concentration of 18 μg per well using Mueller-Hinton agar. Compounds 35a (Ar = 4-NO_2_C_6_H_4_), 35b (Ar = 4-MeC_6_H_4_), 35d (Ar = 4-ClC_6_H_4_), 35e (Ar = 2-ClC_6_H_4_), 35g (Ar = 3-BrC_6_H_4_), 35h (Ar = naphthalen-1-yl), 35i (Ar = 3-OHC_6_H_4_), 35j (Ar = nicotin-4-yl), 35l (Ar = 4-CNC_6_H_4_) and 35m (Ar = 4-(NMe_2_)C_6_H_4_) revealed the most potent activities against *E. coli* species with inhibition higher than 10 mm relative to the result of Ciprofloxacin (13 mm; 5 μg per well). The potency of the compounds 35a–m was recorded with low activities 5–9 mm towards *P. aeruginosa* species. Also, compounds 35a (Ar = 4-NO_2_C_6_H_4_) (15 mm), 35c (Ar = 4-OMeC_6_H_4_) (18 mm), and 35f (Ar = 2,4-Cl_2_C_6_H_3_) (15 mm) showed good activities against *B. subtilis* species compared with the result of Tetracycline (20 mm; 30 μg per well). Compounds 35e (Ar = 2-ClC_6_H_4_) and 35l (Ar = 4-CNC_6_H_4_) are the most potent analogs against *M. luteus* species (20 mm) with high potency than ciprofloxacin (11 mm; 5 μg per well). The electron-withdrawing substituents seemed to be more efficient antibacterial agents than the other substituents on the benzene ring.^105^

**Scheme 27:**
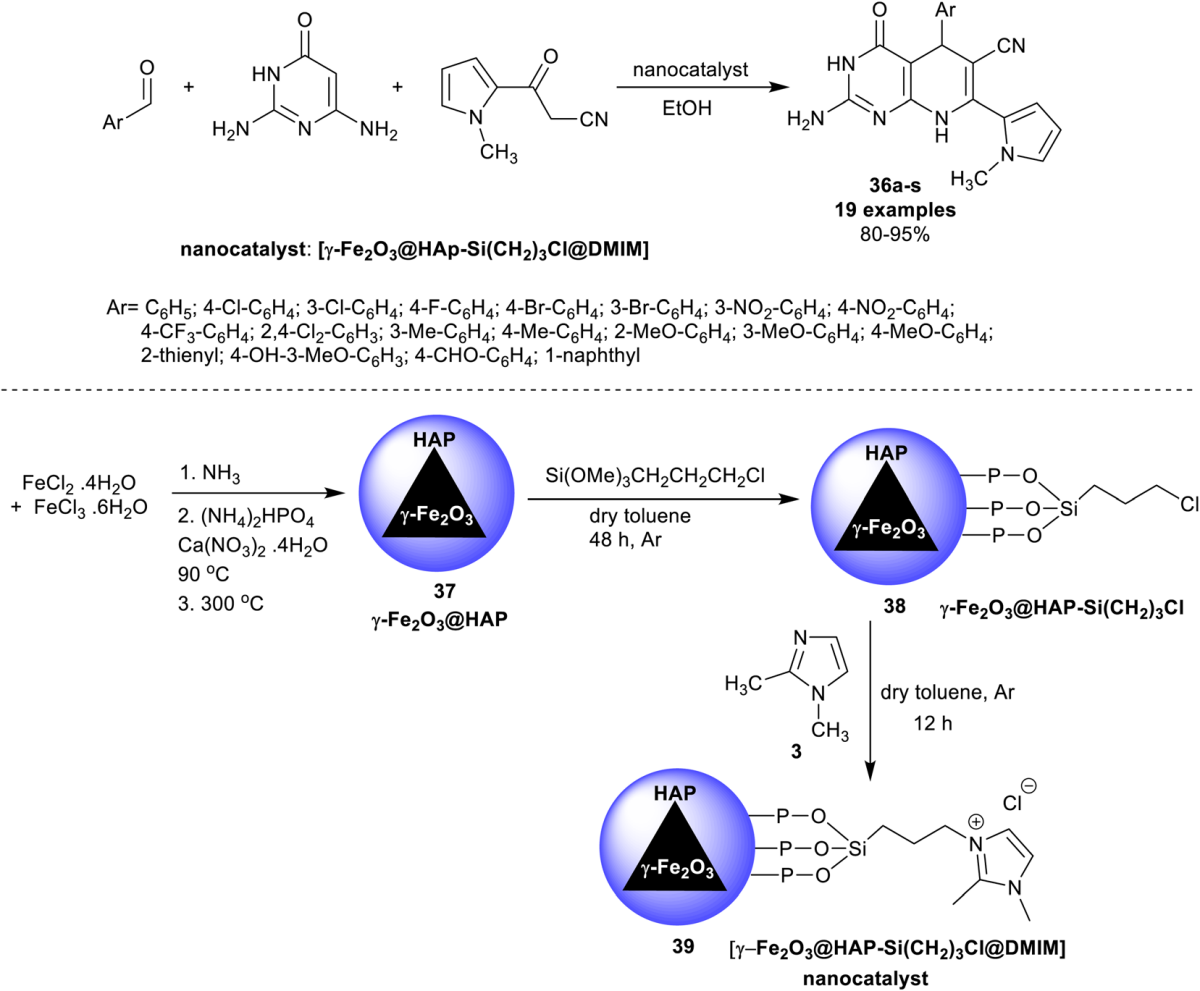
Synthesis of pyrido[2,3-*d*]pyrimidines, and the nano-catalyst.

A series of 5-aryl-7-pyrrolyl-tetrahydropyrido[2,3-*d*]pyrimidine analogs 36a–s were proficiently synthesized Ramezanzadeh *et al.*^107^ using ionic liquid supported on hydroxyapatite-encapsulated γ-Fe_2_O_3_ nano-catalyst. Accordingly, three-component one-pot reactions of aryl aldehydes with diaminopyrimidinone, and 1*H*-pyrrolyl-3-oxopropane-nitrile in ethanol yielded the respective bicyclic products 36. The performance delivered a short reaction time (7–13 min) with the amended yields of the products (80–95%) (Scheme 27). The substituents with electron-withdrawing types are favored for the best product yield, whereas the electron-donating groups led to the formation of the products with reduced yields. The heteroaryl aldehyde and naphthaldehyde led to reduced product yields. For the encapsulation of γ-Fe_2_O_3_ nanoparticles by hydroxyapatite, ferric chloride, and ferrous chloride were treated with ammonia followed by treatment with diammonium phosphate and calcium nitrate under heating conditions. The produced γ-Fe_2_O_3_@HAP 37 solution was treated with (3-chloropropyl)trimethoxysilane in dry toluene to give γ-Fe_2_O_3_@HAP-Si(CH_2_)_3_Cl 38, which reacted with 1,2-dimethyl-1*H*-imidazole to generate the anticipated nano-catalyst 39.

**Scheme 28:**
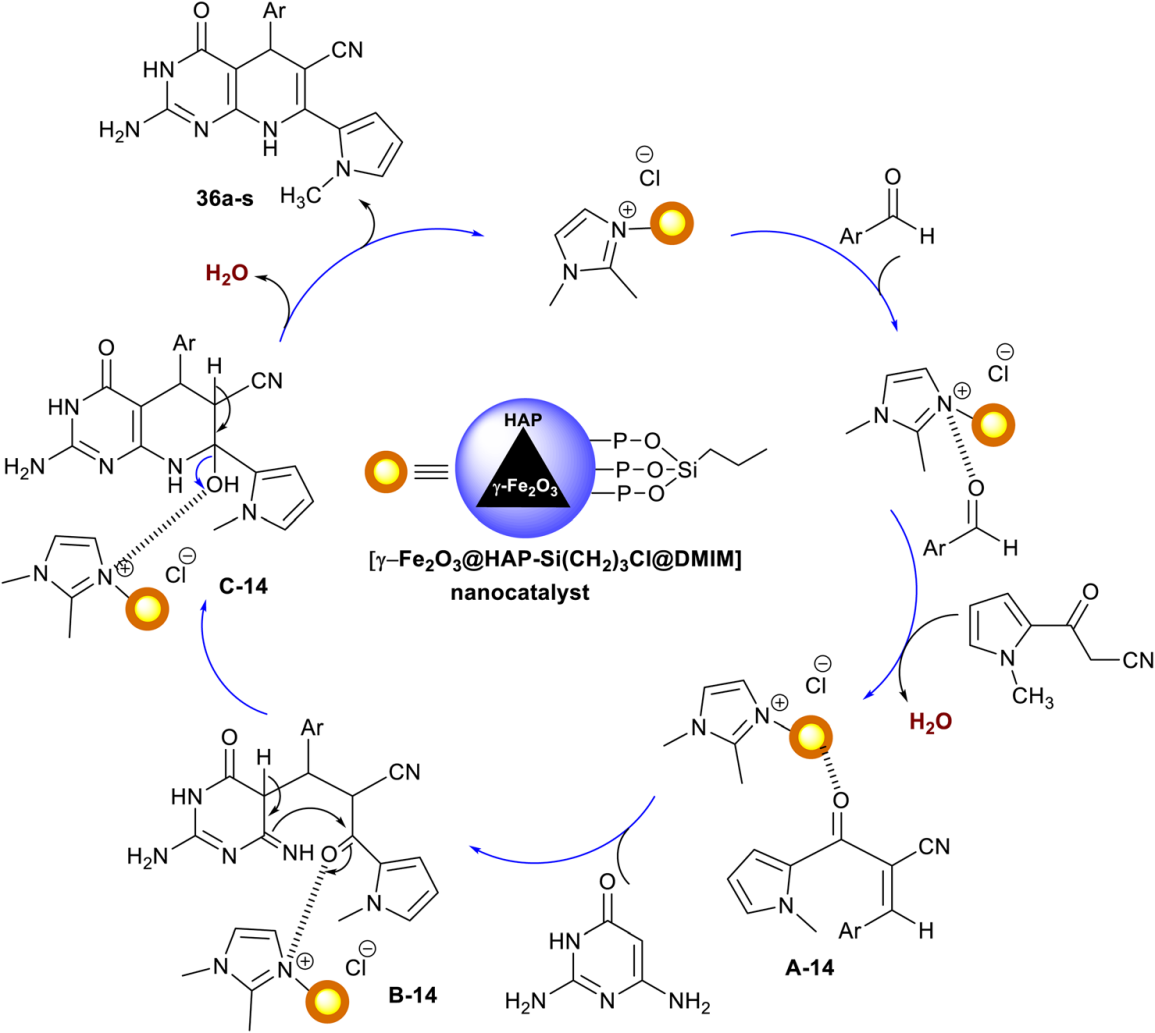
The plausible mechanism for the construction of pyrido[2,3-*d*]pyrimidines.

The ionic liquid supported on hydroxyapatite-encapsulated nano-catalyst activated the aldehyde carbonyl functional group of the aryl aldehydes for the condensation with 3-(1-methyl-1*H*-pyrrol-2-yl)-3-oxopropanenitrile. The formed arylidene intermediate A-14 interacted with 2,6-diaminopyrimidin-4(3*H*)-one through Michael's addition type reaction to generate the intermediate B-14. Cyclocendensation of the intermediate B-14 produced the intermediate C-14, in which the release of the nano-catalyst afforded the bicyclic products in the form of tetrahydropyrido[2,3-*d*]pyrimidines 36 (Scheme 28). The release of the nano-catalyst allowed its reusability for several times, but the work did not provide the method for the separation of the nano-catalyst from the reaction mixture.^107^

**Scheme 29:**
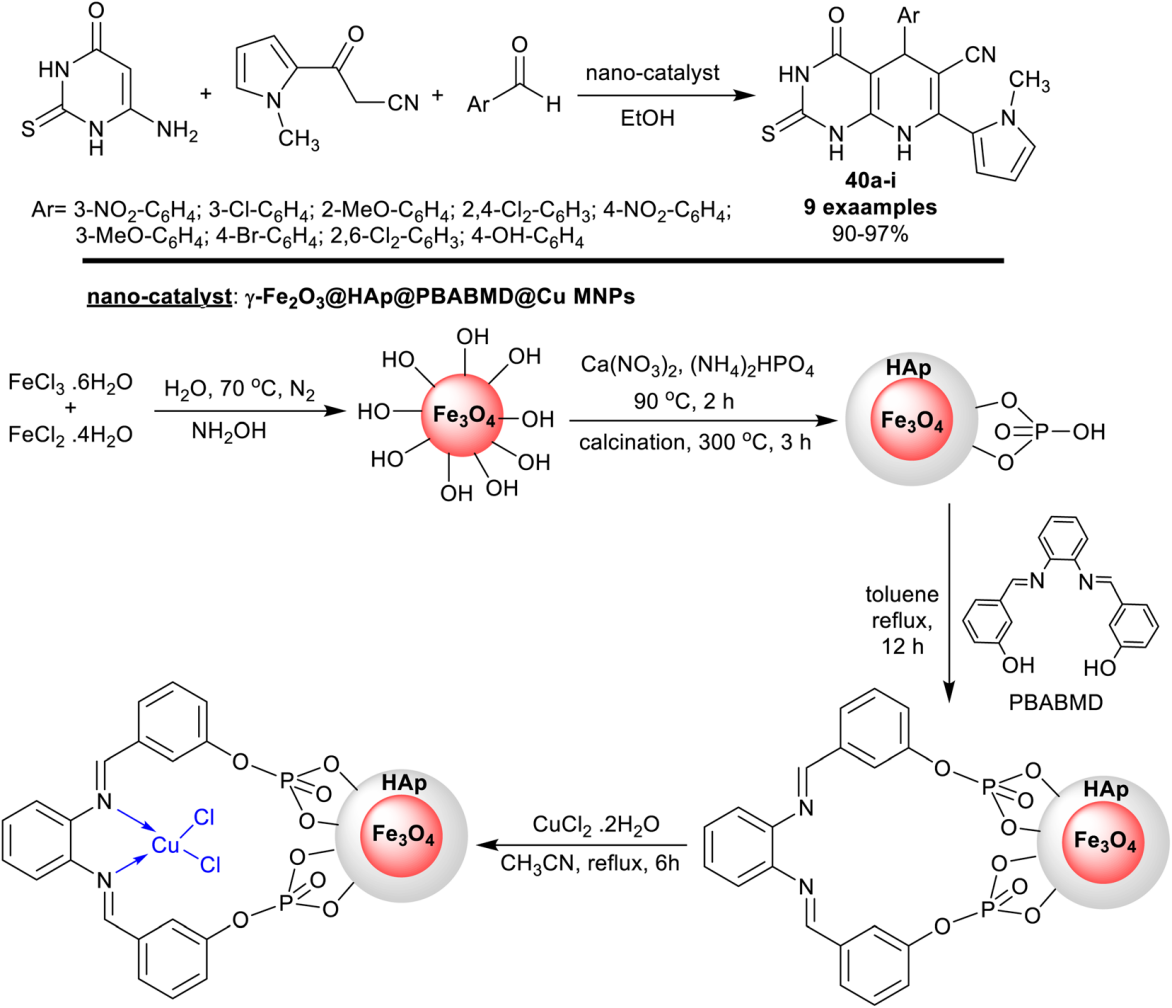
Synthesis of pyrrolyl-hexahydropyridopyrimidines, and the nano-catalyst.

Recently, Saberikhah *et al.*^108^ have developed the utility of the complex of Cu(ii)-PBABMD restrained on magnetic core–shell of γ-Fe_2_O_3_@HAp for the synthesis of 1-methyl-1*H*-pyrrolyl-hexahydropyrido[2,3-*d*]pyrimidine analogs 40a–i (Scheme 29). Therefore, the reaction sequence is a one-pot technique involving the interactions of three-component of substrates, *e.g.* 6-amino-thiouracil reacted with 1-methyl-2-cyanoacetyl-1*H*-pyrrole, and aryl aldehydes in ethanol containing the nano-catalyst gave the predicted products in brilliant yields 90–97% after short reaction times (8–13 min). The reactions were run in ethanol under heating and nano-catalytic conditions. The recyclability of the catalyst, along with its extraordinary productivity added relevance to this procedure owing to the multi-step sequence of the nano-catalyst preparation. The incorporation of the pyrrole ring into the bicyclic pyridopyrimidines is beneficial for the future development of the biological potency of these compounds. The highest overall yield was recorded for the 3-nitro substituent on the phenyl ring (97%), while the least yield was recorded for the 4-hydroxy substituent (90%) preferring generally the chlorine and bromine substituents on the phenyl ring.

**Scheme 30:**
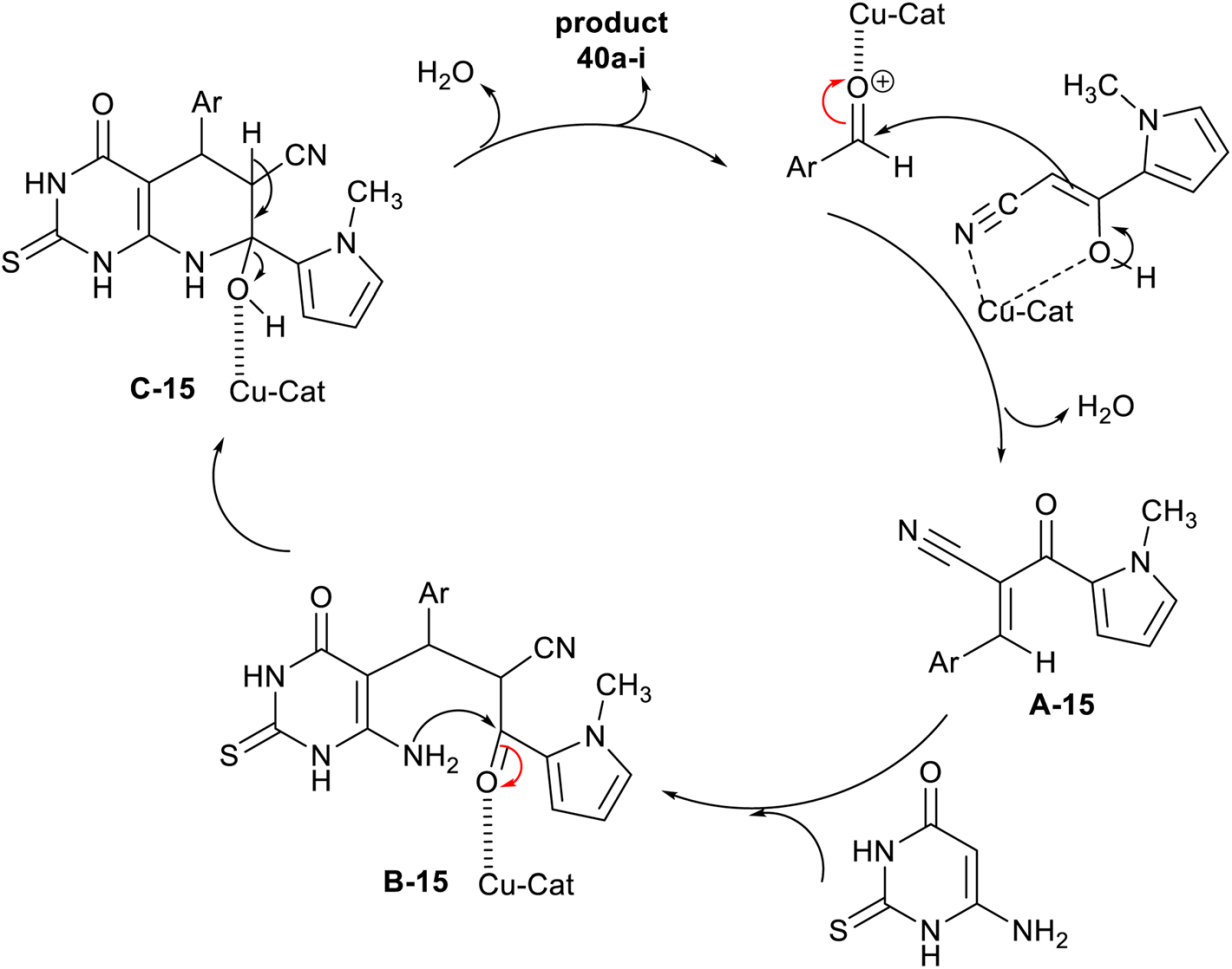
The conceivable mechanistic route for the synthesis of pyrrolyl-bicyclic systems.

The plausible mechanism of the synthesis regarding pyrrolyl-hexahydropyridopyrimidines 40a–i is shown in Scheme 30. First, the nano-catalyst activated the carbonyl group of the aldehydes in the step of Knoevenagel condensation with an active methylene group of cyanoacetyl pyrrole involving the formation of two C=C bonds. The arylidene intermediated interacted with 6-amino-thiouracil by the Michael addition step sequence followed by intramolecular cyclization with the formation of a C–N bond and condensation to give the bicyclic pyridopyrimidines 40a–i. Generally, the pyridine ring closure involved the formation of one C–N bond and two C–C bonds.^108^

**Scheme 31:**
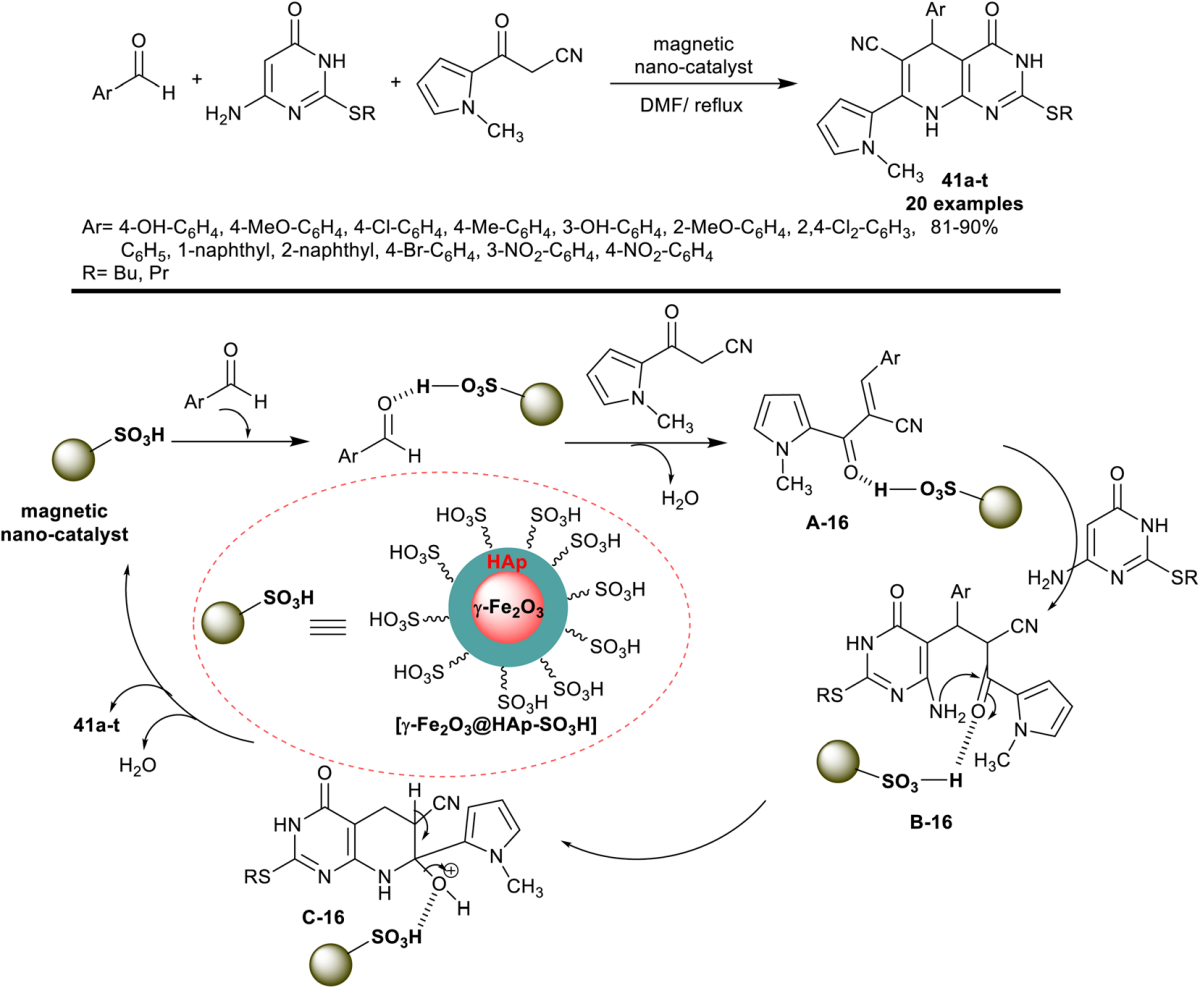
A schematic mechanism for the synthesis of pyrrolyl-tetrahydro-pyridopyrimidines.

In 2018, Jahanshahi *et al.*^33^ developed the synthesis of 2-(alkylthio)-7-(*N*-methyl-1*H*-pyrrolyl)-tetrahydro-pyridopyrimidine derivatives 41a–t under nano-catalytic conditions using [γ-Fe_2_O_3_@HAp-SO_3_H]. Accordingly, three-component reactions of aryl aldehydes with thioalkyl-aminouracil, and 3-(1-methyl-1*H*-pyrrol-2-yl)-3-oxopropanenitrile in DMF containing the nanocatalyst at reflux temperature gave the desired heteroaryl-bicyclic products 41a–t in a one-pot procedure. The encapsulation of the γ-Fe_2_O_3_ nanoparticles with hydroxyapatite and sulfonic acid moiety with acidic functionality enabled the Knoevenagel condensation, Michael addition, pyridine ring cyclization, and condensation steps in the reaction sequence (Scheme 31). The advantage of this method was found in the ease of the nanocatalyst preparation, recyclability, improved product yields, and purity, and short reaction time (7–14 min).

The antibacterial activity of compounds 41a–t (Scheme 31) was assessed against *M. luteus*, *B. subtilis*, *E. coli*, and *P. aeruginosa* bacterial species. Ciprofloxacin and tetracycline were applied as standard antibiotics. Most of the compounds demonstrated potent antibacterial activities with inhibition zones greater than 7 mm. Compounds 41b (Ar = 4-MeOC_6_H_4_), 41f (Ar = 2-MeOC_6_H_4_), 41g (Ar = 2,4-Cl_2_C_6_H_3_), 41i (Ar = 1-naphthyl), 41j (Ar = 2-naphthyl), 41p (Ar = 3-O_2_NC_6_H_4_) and 4q (Ar = 4-MeOC_6_H_4_) are inactive agents against *E. coli* species. Compound 41s (Ar = 2-MeOC_6_H_4_) is extremely effective against *E. coli* species (11 mm; 18 μg per well) relative to ciprofloxacin (13 mm; 5 μg per well). Compounds 41o (Ar = 4-MeC_6_H_4_) and 41t (Ar = 4-OHC_6_H_4_) are the most potent against *P. aeruginosa* species (15 mm; 5 μg per well). Also, compounds 41c (Ar = 4-ClC_6_H_4_) and 41g (Ar = 2,4-Cl_2_C_6_H_3_) were extremely effective activities against *B. subtilis* species (18 mm; 5 μg per well), while compounds 41k (Ar = 2,4-Cl_2_C_6_H_3_) and 41l (Ar = 4-ClC_6_H_4_) were the most potent analogs against *M. luteus* species (22 and 20 mm, respectively). The potential activity of the compound is dependent on the introduction of the bicyclic pyridopyrimidine ring, thioalkyl moiety, and the nature of substituents on the benzene ring, as well as the type of the assessed microorganism.^33^

### 2.3 Four-component synthesis

**Scheme 32:**
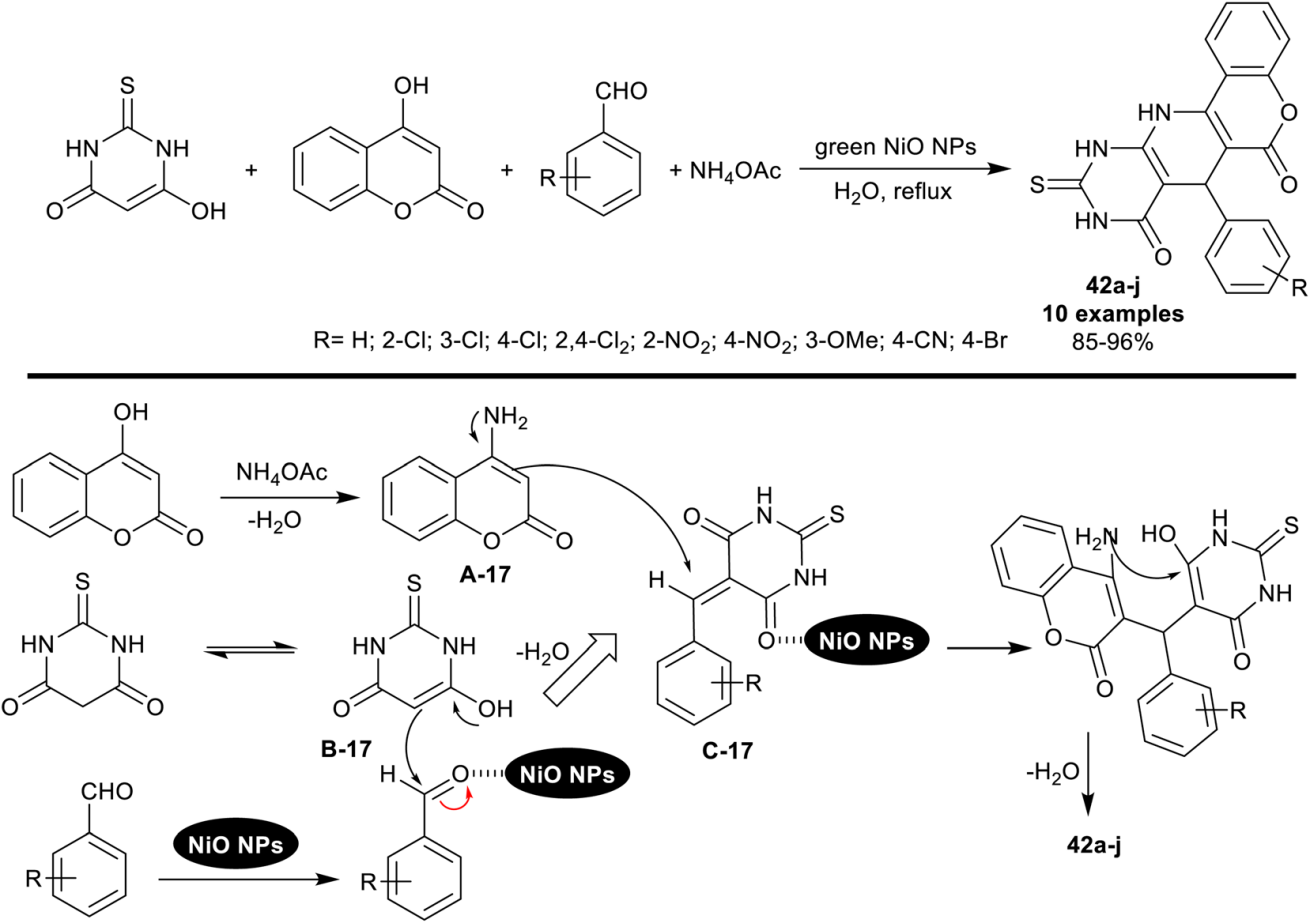
Synthesis of pyridopyrimidines using nickel oxide nanocatalyst.

An innovative procedure was reported by Moavi *et al.*^109^ deliberating the utility of marine macroalgae extract for the biosynthesis of NiO nanoparticles. The NiO NPs were used as an effectively applicable nanocatalyst for the synthesis of a series of pyridopyrimidines 42a–j (Scheme 32) through four-component one-pot reactions. The reactions were run in water as a green solvent under nano-catalytic, and heating conditions. The characteristic feature of this procedure is to provide an appropriate green technique to prepare pyridopyrimidines with developed yields.

Under green optimized conditions, the catalytic NiO nanoparticles were efficiently applied using 5 mol% to obtain the best yield from the tetracyclic products 42a–j by reactions of the one-pot four-component reactions of equimolar rations of thiobarbituric acid, aldehydes, ammonium acetate, and 4-hydroxy-2*H*-chromen-2-one. The recyclable nickel oxide nanocatalyst was prepared by mixing a solution of NiCl_2_·6H_2_O with marine red algae extract under gentle heating and stirring conditions. The plausible mechanism for the bicyclic ring construction is shown in Scheme 32, which indicated the amination of 4-hydroxy-2*H*-chromen-2-one by interaction with ammonium acetate, enolization of thiobarbituric acid, formation of the arylidene intermediates, and cyclocondensation steps. The nanocatalyst activated all the reaction steps and released from the reactions in the final step to be reused. The aryl ring of aldehydes substituted with electron-withdrawing groups or atoms led to the highest product yields from this sequence of reactions (≈>90%) than the unsubstituted or aryl aldehydes substituted with electron-donating groups.^109^

### 2.4 Synthesis of spirocyclic systems

**Scheme 33:**
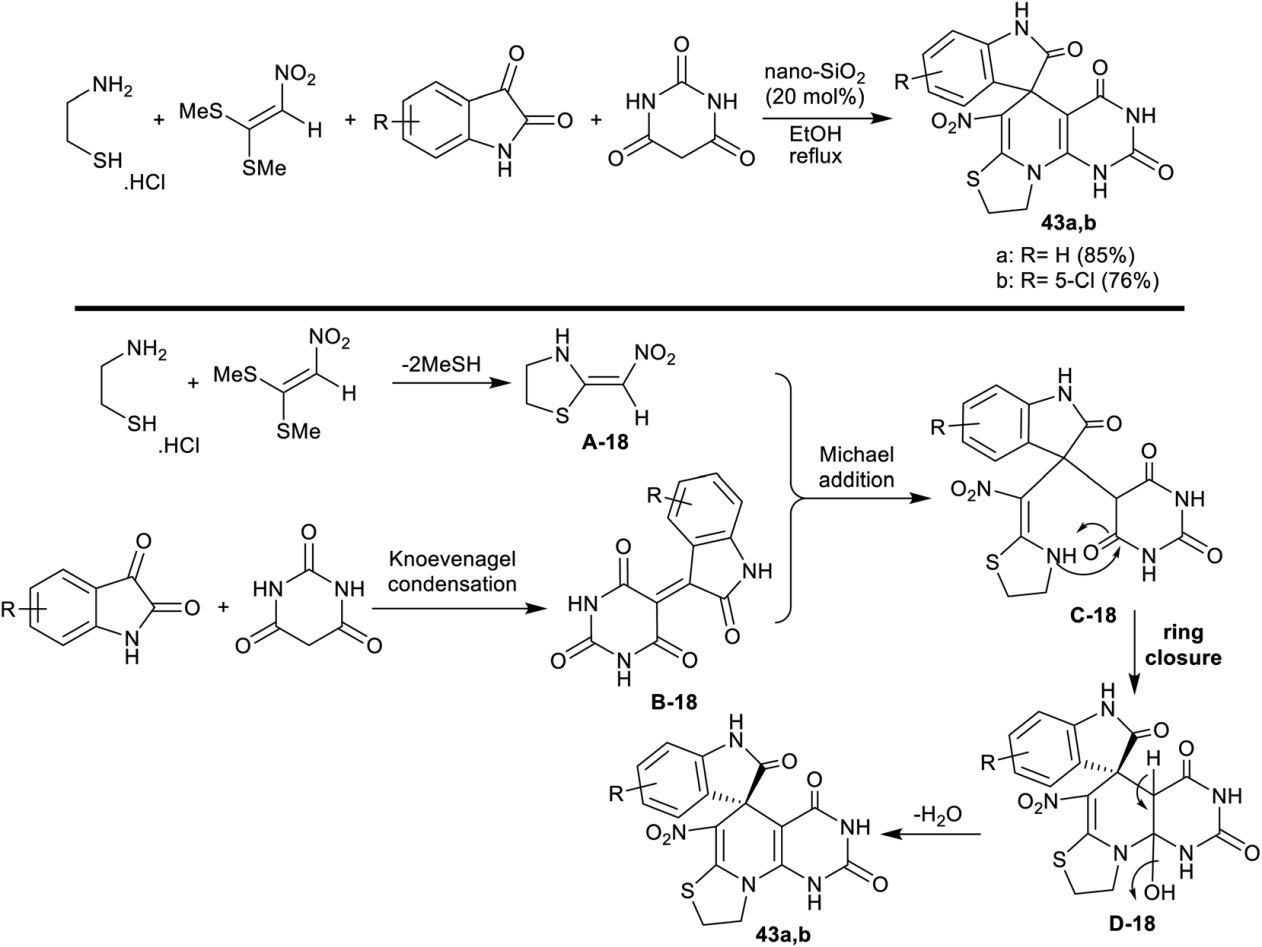
Synthesis of spiro[indoline-thiazolopyridopyrimidine] derivatives.

Nasri *et al.*^110^ developed a domino four-component one-pot procedure for the synthesis of spiro[indoline-thiazolopyridopyrimidine] analogs 43a,b under nano-SiO_2_ (20 mol%). Thus, the reactions of 2-aminoethane-1-thiol hydrochloride with (2-nitroethene-1,1-diyl)bis(methylsulfane), isatins, and barbituric acid in ethanol at their reflux temperature under nano-SiO_2_ catalytic conditions to yield the target products (Scheme 33). The nano-catalyst acts as a Lewis acid for the promotion of the reacted substrates to produce the products in good yields.

The sequence of this reaction-type was postulated as 2-aminoethane-1-thiol hydrochloride reacted with (2-nitroethene-1,1-diyl)bis(methylsulfane) to generate the intermediate A-18. The intermediate B-18 was formed by Knoevenagel condensation between barbituric acid, and isatins. Consequently, the intermediates A-18 and B-18 followed a Michael addition step with the generation of the intermediate C-18. The cyclization of the intermediate C-18 followed by condensation produced the spirocyclic products 43a,b. The nano-catalyst is recyclable with reduced toxicity, low-cost materials, and ease of the product workup, whereas the procedure is suitable for the preparation of drug-like molecules applying succeeding progress (Scheme 33).^110^

**Scheme 34:**
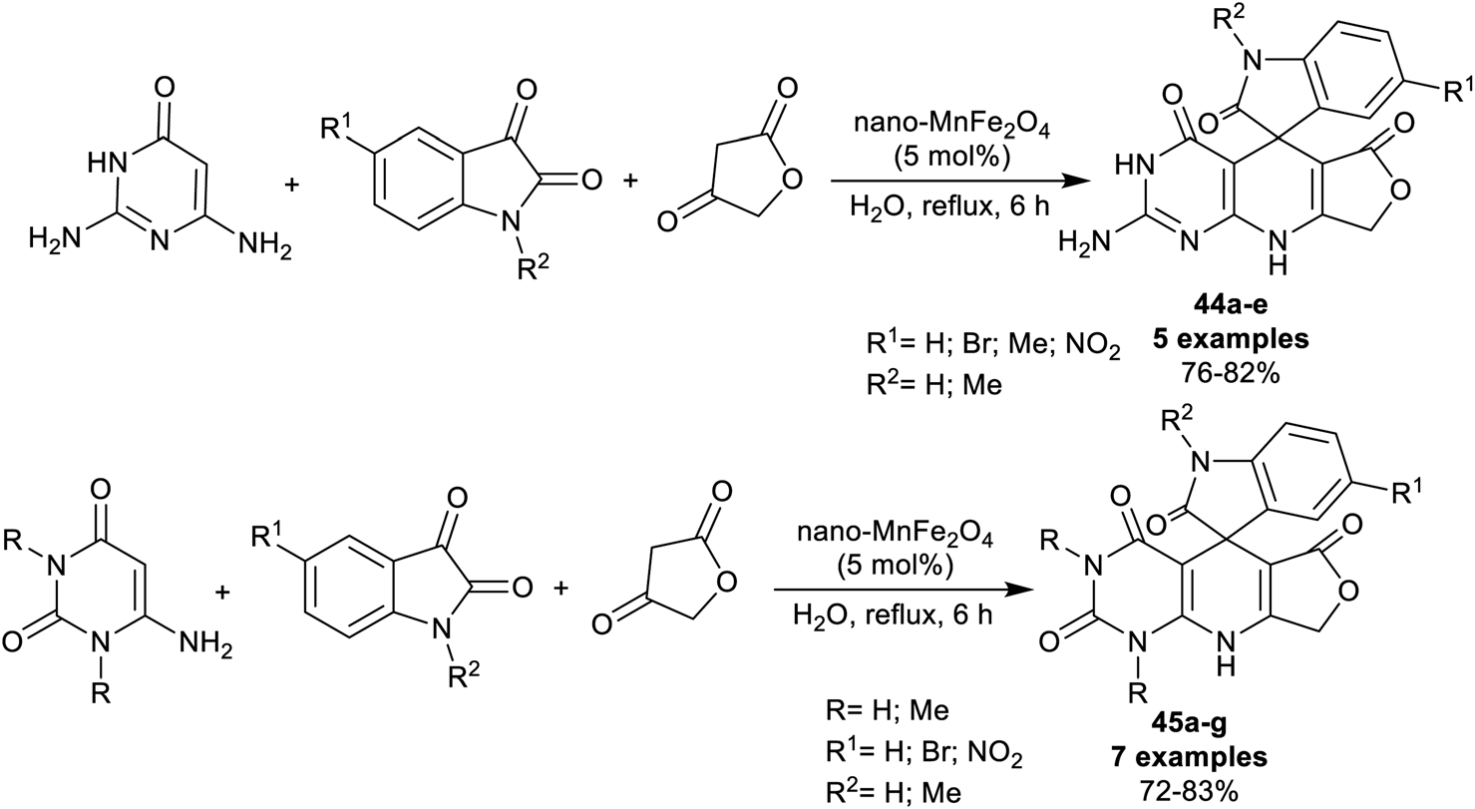
Synthesis of spiro[furopyrido[2,3-*d*]pyrimidine-5,3′-indoline] derivatives.

The spiro-oxindole motifs are benefitted heterocycles as these compounds are under the categories of alkaloids and natural products,^111^ with stimulating structural sorts and extensive assortment of beneficial bioactive, and pharmacological characteristics.^112–116^ In 2014, Naeimi *et al.*^117^ synthesized a series of spiro[furopyrido[2,3-*d*]pyrimidine-5,3′-indoline] derivatives 44a–e and 45a–g under nano-catalytic conditions using manganese ferrite nanoparticles. Subsequently, multicomponent one-pot synthesis was also applied through reactions of 2,6-diaminopyrimidine-4(3*H*)-one or uracil derivatives with isatin derivatives and tetronic acid in water at reflux temperature to furnish the target spirocyclic compounds 44a–e and 45a–g (Scheme 34). The challenges that face the research organic synthesis are to find suitable solvents with nontoxic action and to explore a distinct recyclable nano-catalyst for improving the product yields of the reactions. The method presented a green protocol, suitable one-pot process, nano-catalyst reusability by magnetic process, improved product yields, high reaction rate, and ease of product separation. The use of MnFe_2_O_4_ nanocatalyst produced an improved yield of compound 44a (R_1_ = R_2_ = H; 82%) than the use of nano CuFe_2_O_4_ (74%) with the same reaction rate. Manganese ferrite nanoparticles were prepared by co-precipitation of manganese(ii) chloride, and iron(iii) chloride (1 : 2 molar ratio) in water containing sodium hydroxide solution with continuous stirring under heating conditions.

**Scheme 35:**
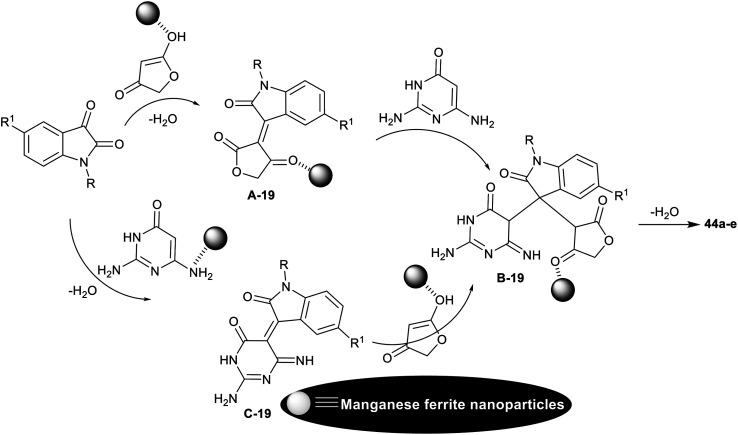
The plausible mechanistic route for the synthesis of spiro[furopyrido[2,3-*d*]pyrimidine-5,3′-indolines].

The plausible mechanistic route for the synthesis of spirocyclic compounds 44a–e is outlined in Scheme 35. Particularly, the tautomerization of tetronic acid was accomplished by the action of the nano-catalyst for condensation reactions with isatins through a swift Knoevenagel condensation step to generate the arylidene intermediates A-19. The interaction of the intermediates A-19 with 2,6-diaminopyrimidine-4(3*H*)-one by the aid of the nano-catalyst activation produced the intermediates B-19. Cyclocondensation of the intermediates B-1 through intramolecular nucleophilic attack of the imino group at the activated carbonyl group gave the target spirocyclic products. In another route, the condensation step was accomplished between isatins and 2,6-diaminopyrimidine-4(3*H*)-one to generate intermediates C-19, which interacted with the activated tetronic acid to achieve the pyridine ring closure to give the anticipated products 44a–e.^117^

### 2.5 Arylation reactions

**Scheme 36:**
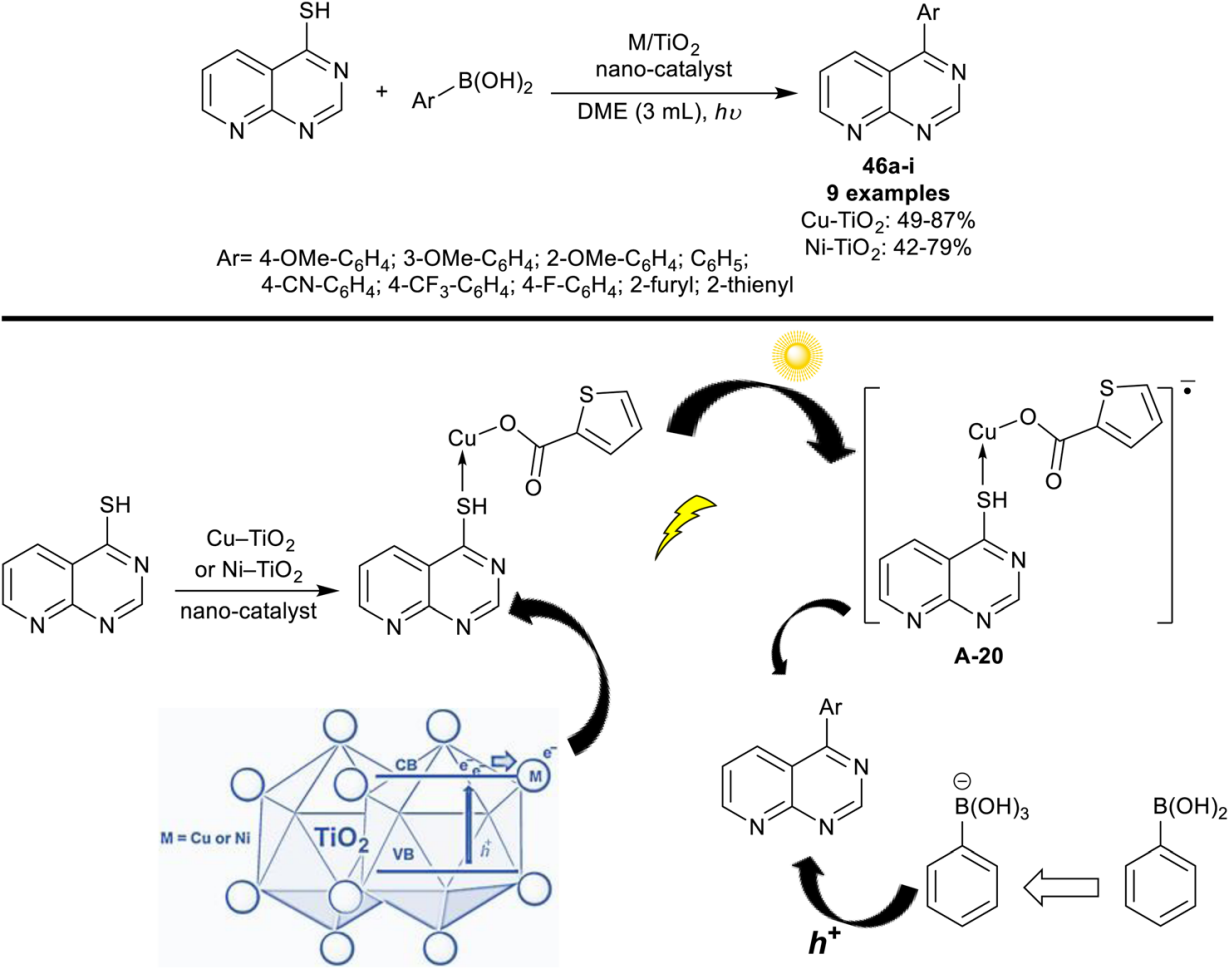
Photocatalytic arylation of pyrido[2,3-*d*]pyrimidine-4-thiol.

Riadi *et al.*^118^ have progressed the arylation of pyrido[2,3-*d*]pyrimidine-4-thiol through nanostructured titanium dioxide doped with nickel and copper. The nano-catalyst was prepared by a sol–gel procedure. The spectral analysis of the nano-catalyst indicated a rutile phase, and biphasic anatase-rutile structures for Cu- and Ni-doped titanium dioxide, respectively. Thus, Liebeskind–Srogl reactions of pyrido[2,3-*d*]pyrimidine-4-thiol with aryl boronic acid under nano-catalyst, and UV radiance conditions were investigated. The products, 4-arylpyrido[2,3-*d*]pyrimidines 46a–i obtained slightly higher overall yields using the Cu-doped TiO_2_ nano-catalyst compared to the Ni-doped TiO_2_ catalyst (Scheme 36).

The mechanism postulated for this reaction sequence is shown in Scheme 36. Eventually, under the optimized UV radiance, the electrons transferred from the valence band of titanium dioxide to its transmission band, creating an electron–hole pair. Regrettably, a rapid combination of this electron–hole pair within the titanium dioxide semiconductor led to no participation of the photo-excited electrons in the catalytic reaction. In addition to its potential to reduce the band gap, the recombination of electron–hole was reduced by the action of the doped solution through the trapped excited electrons when the pure metal is loaded over the surface of the nanoparticles of titanium dioxide or by heterojunction in the case of metal oxide settling on the structure of titanium dioxide. Thus, the interaction tended to the separation of electron–hole, reduced recombination, and enhanced the interfacial charge transfer. It was perceived that the reaction was not implemented without the Cu–TiO_2_ co-catalyst even using a metal-doped TiO_2_ catalyst under UV irradiation. Therefore, the first step reaction involved the interaction of Cu–TiO_2_ with pyrido[2,3-*d*]pyrimidine-4-thiol forming a complex that seized photogenerated electrons. The C–B bond of aryl boronic acids was activated by the formed holes and thus transformed into B(OH)_3_^−^ by attracting an OH^−^ from the base medium of the reaction. Lastly, the cross-coupling reaction occurred between the oxidized aryl boronic acids, and the thiol to give the target products through transmetalation and reductive elimination.^118^

## 3 Synthesis of annulated pyrido[1,2-*a*]pyrimidines

Heterocyclic pyrido[1,2-*a*]pyrimidine is the basic scaffold of certain drugs comprising pemirolast (antiasthmatic agent),^119^ pirenperone (tranquilizer),^120^ and barmastine,^121^ (antiallergic agent). As a concern, numerous approaches have been adopted for the synthesis of 4*H*-pyrido[1,2-*a*]pyrimidine analogs.^122^ Nevertheless, these procedures have several disadvantages, for instance, the use of extremely toxic and destructive reagents, volatile solvents, metallic catalysis, critical conditions of the reactions, or the formation of unsafe wastes. Besides, the most practical disadvantage of present approaches is that the consumption of the catalyst through the reaction, and cannot be recycled thus decreasing the turnover rate, which is an imperative limitation from an industrial theme.

**Scheme 37:**
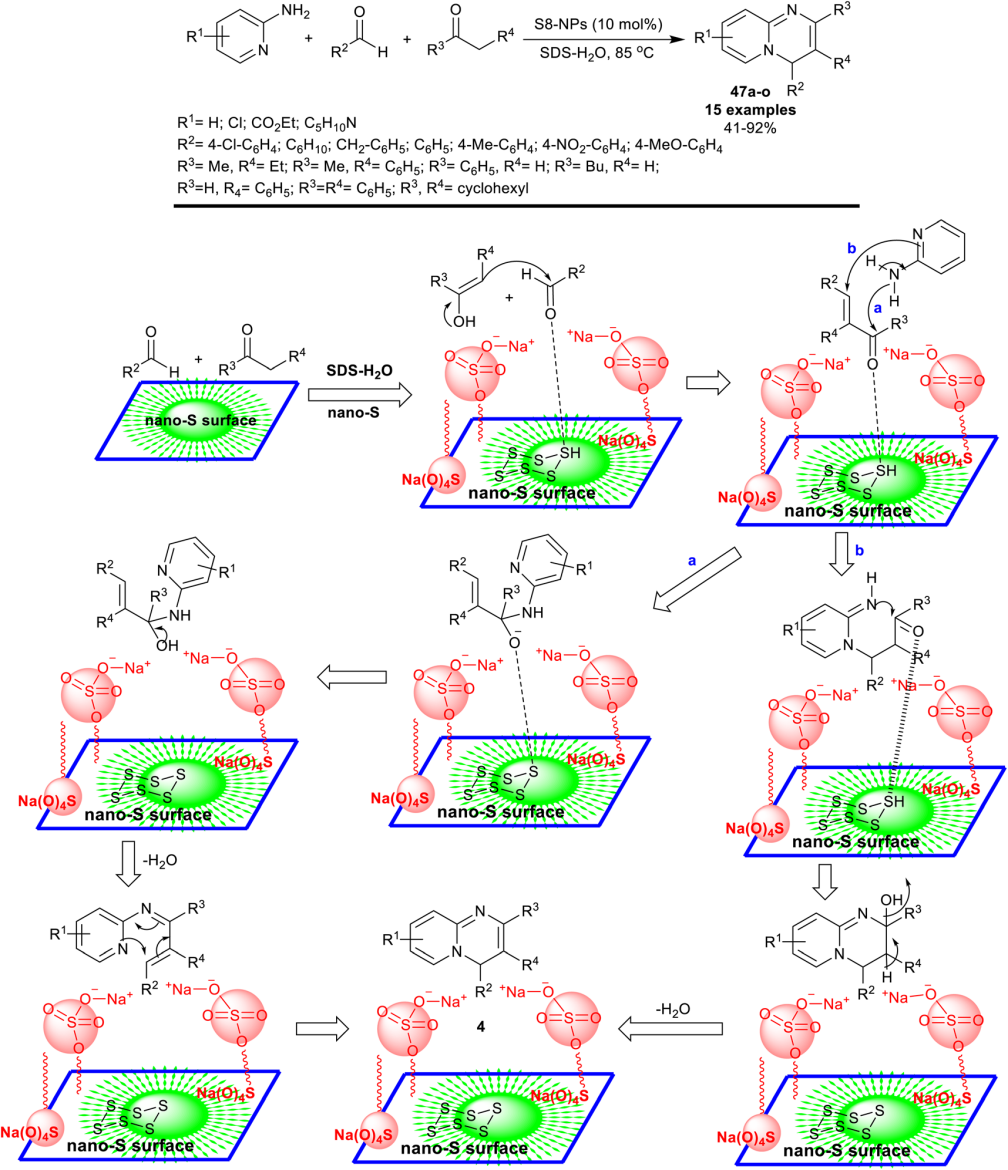
Schematic synthesis, and the conceivable mechanism of 4*H*-pyrido[1,2-*a*]pyrimidines.

Owing to intensifying apprehensions about the toxicity of organic solvents and their poisonous performance on human health and the ecosystem, the utility of green solvents such as water has recently generated extensive attention.^123^ The ideal solvents such as water were included in many organic reactions as they are economical, non-toxic, and green solvents. A series of 4*H*-pyrido[1,2-*a*]pyrimidines 47a–o were synthesized by Sagir *et al.*^124^ under nano-catalytic conditions using an admicellar system supported by sulfur nanoparticles in the presence of surfactant such as SDS (Scheme 37).

Therefore, three-component reactions of 2-aminopyridines with aryl aldehydes, and active methylene components under the elevated conditions yielded the anticipated 4*H*-pyrido[1,2-*a*]pyrimidines 47a–o. SDS was used to increase the solubility of the organic substrates, in which it was adsorbed on the surface of the nanoparticles at a lower concentration of the surfactant. The preparation of S8-NPs was attained by heating the elemental sulfur at 120 °C, cooling it to room temperature, transferring it over an ice bath, and grinding it with mortar and pestle. The process involved the catalytic conversion of hydrogen sulfide.^125^ The enolization of active methylene compounds was supported by the nano-catalyst for Knoevenagel condensation with aldehydes to form the arylidene intermediates. Therefore, two possible routes (a and b) are proposed for the interaction of the arylidene intermediates with 2-aminopyridines. Route “a” involved a nucleophilic attack of the exocyclic amino group at the carbonyl group of the arylidene intermediate and subsequent intramolecular cyclization. Route “b” involved a nucleophilic attack of the nitrogen atom of the pyridine ring at the terminal carbon of the arylidene intermediate through the Michael addition sequence. Both routes were next tended to intramolecular cyclization with the support of the S8-NPs–SDS system (Scheme 37).^124^

**Scheme 38:**
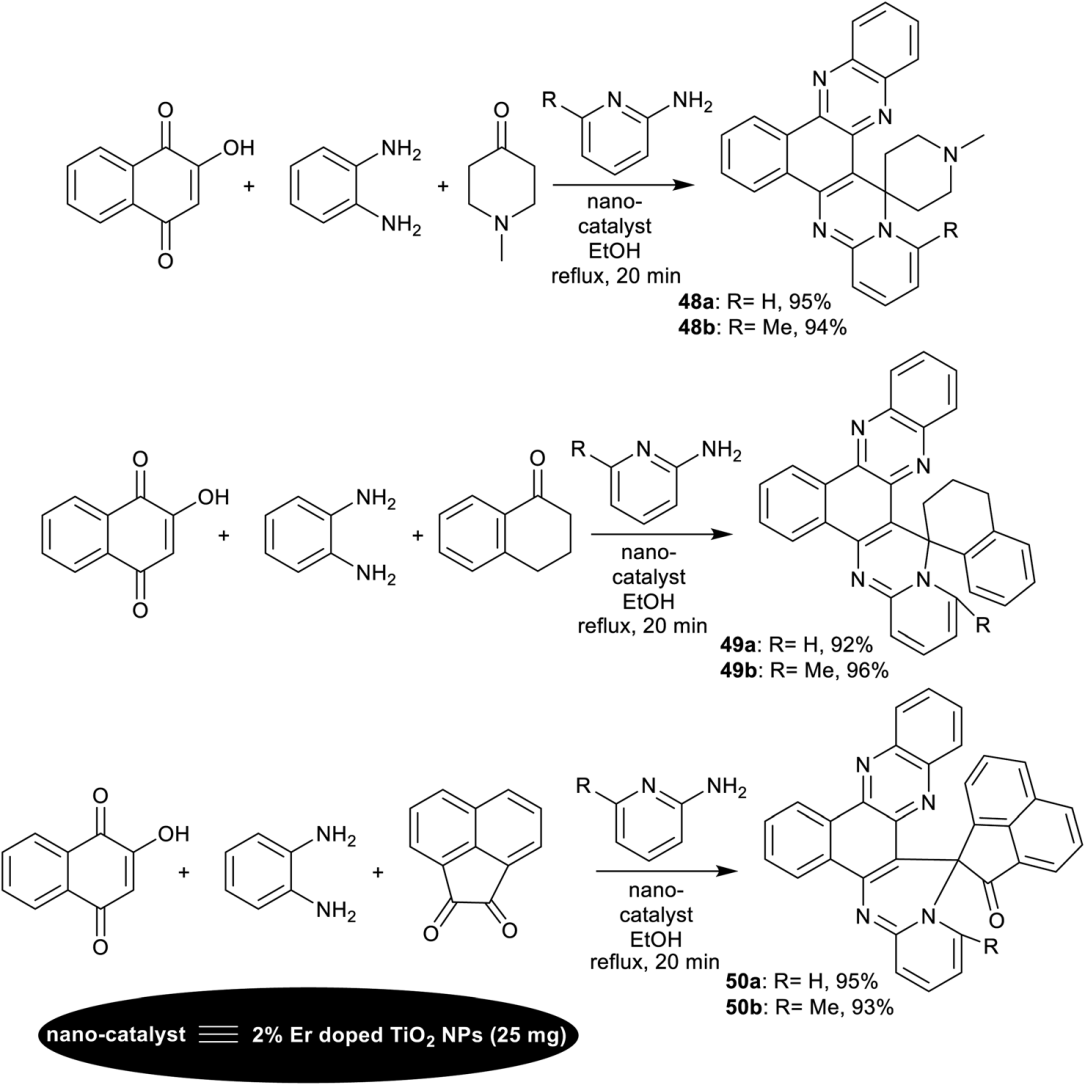
Four-component synthesis of spiro[benzopyrido[1′,2′:1,2]pyrimidophenazine] derivatives.

Verma *et al.*^126^ have intended the synthesis of spiro-annulated-polycyclic systems integrated pyridopyrimidine skeleton 48–50a,b. In particular, four-component reactions in a one-pot procedure were applied under nano-catalytic conditions using erbium-doped titanium dioxide nanoparticles. In this course, reactions of 2-hydroxynaphthalene-1,4-dione with *o*-phenylene-diamine, active methylene compounds, and *o*-aminopyridines under the optimized conditions gave the target compounds (Scheme 38). The green and supportable domino procedure produced exceptional yields of the polycyclic products (92–96%) along with the conceivable nano-catalyst reusability. The nano-catalyst performed as a heterogeneous acid catalyst that supported the reaction mechanism sequence and increases the reaction rate in all steps to construct the final product. No reaction proceeded under either catalyst-free or solvent-free conditions. The efficient amount of the nano-catalyst was found as 25 mg, which equals the nano-catalyst efficiency using 30 mg. The doping of erbium with titanium dioxide impacted the nano-catalyst efficiency to improve the reaction rate, and further increase the product yield. The nano-catalyst, Er-doped TiO_2_ NPs was prepared by the sol–gel method from titanium tetra-isopropoxide in a mixture of water, ethanol, and acetic acid. ErCl_3_ solution was prepared in a mixture of water and ethanol (1 : 10) and subsequently added to the previously prepared solution with the dropwise addition of 1-thioglycerol.

**Scheme 39:**
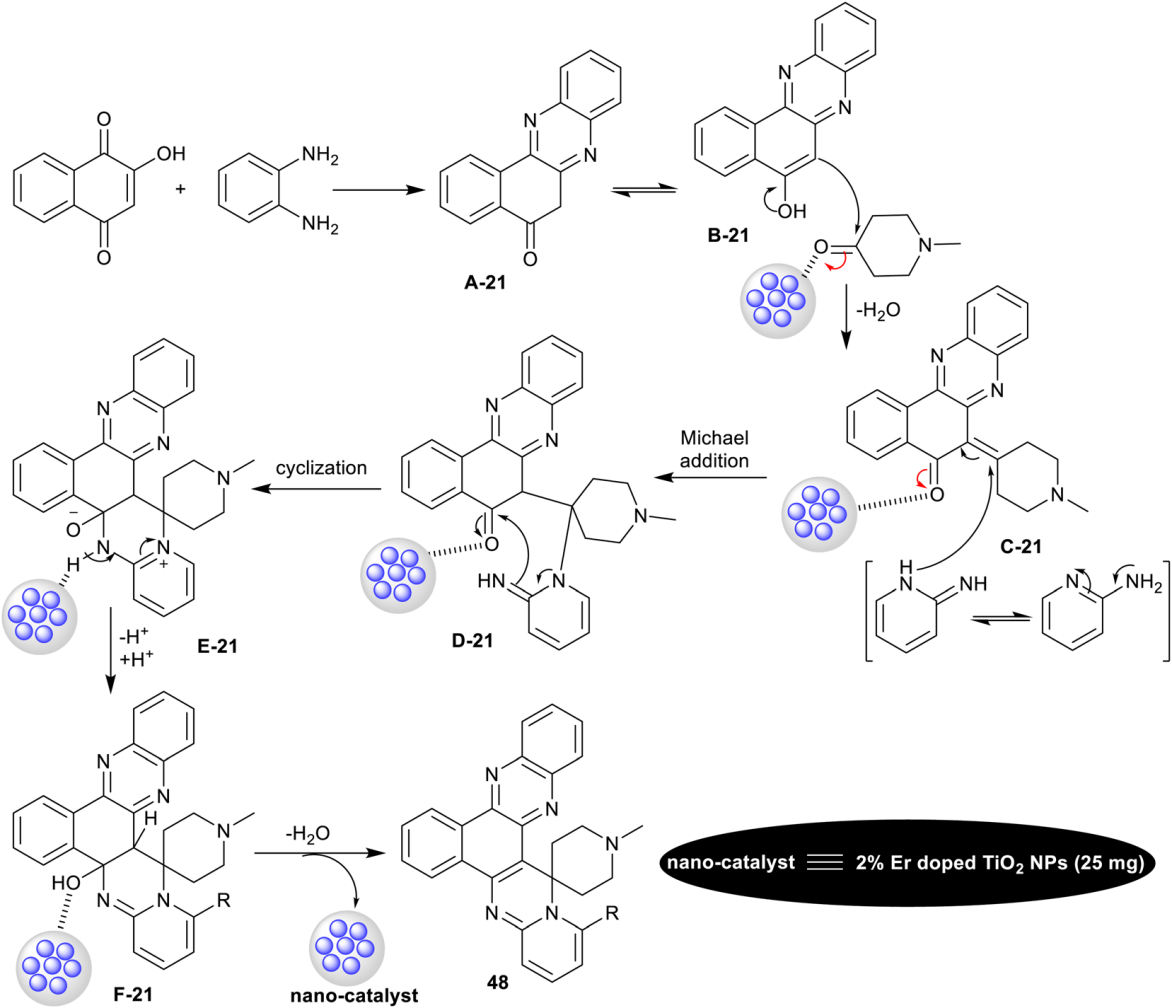
The postulated mechanism for the synthesis of spiro-annulated-polycyclic heterocycles.

The probable mechanistic route for the synthesis of the spiro-annulated-polycyclic heterocycles 48 is shown in Scheme 39. In the first step, 2-hydroxynaphthalene-1,4-dione reacted with *o*-phenylene-diamine to generate the intermediate A-21. The enolization of intermediate A-21 generated intermediate B-21, which interacted with active methylene compounds with the support of the nano-catalyst to form the arylidene intermediate C-21. Subsequently, Michael's addition of the generated intermediate C-21 with *o*-aminopyridines formed the intermediates D-21. Intramolecular cyclization of the intermediates D-21 led to pyrimidine ring cyclization “intermediate E-21”. The nano-catalyst supported the proton transfer, and subsequent condensation to give the final products with the release of the nano-catalyst and further recycled.^126^

**Scheme 40:**
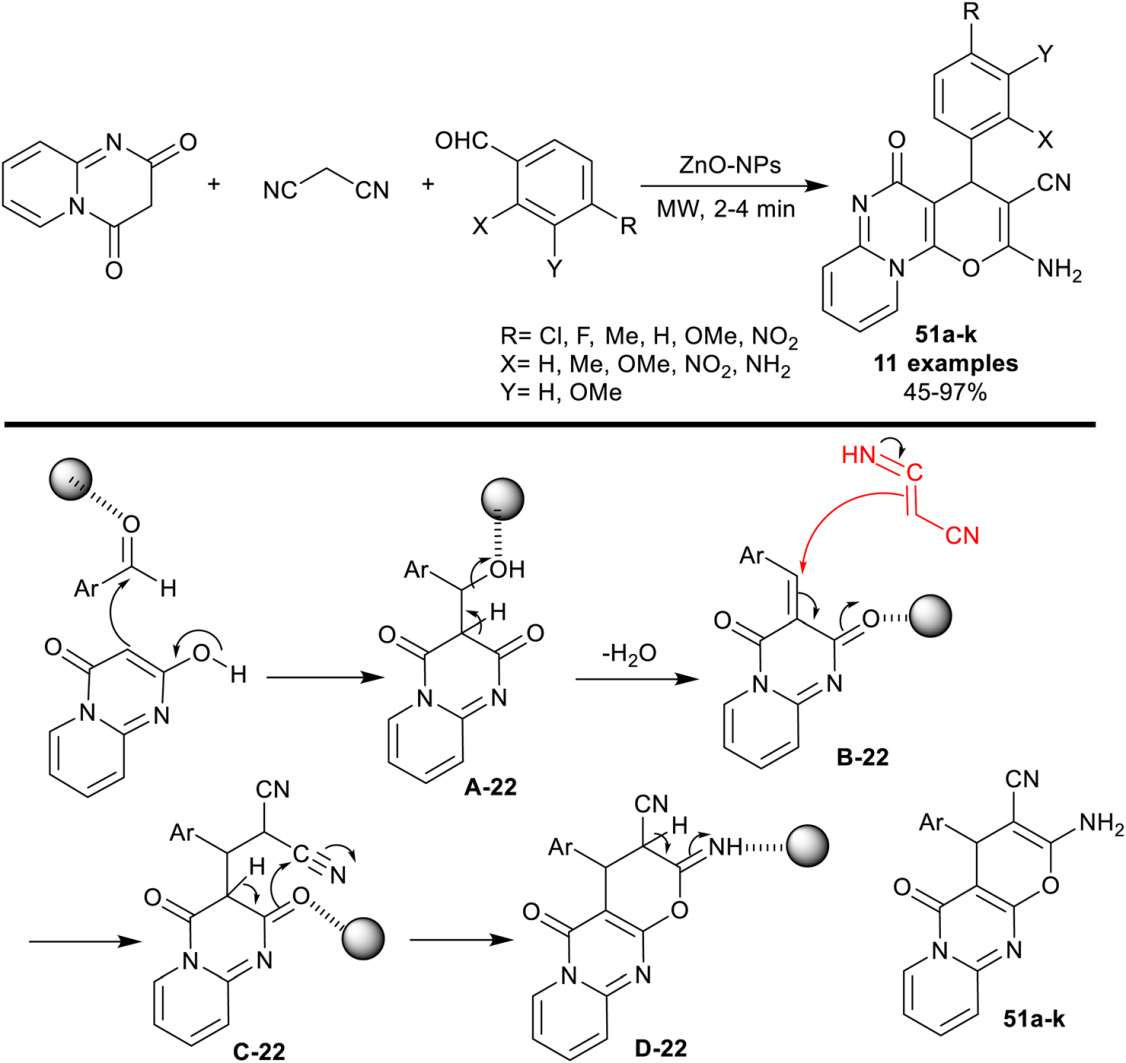
Three-component synthesis of pyranopyrido[1,2-*a*]pyrimidines.

A series of pyranopyrido[1,2-*a*]pyrimidines 51a–k were synthesized through three-component reactions of malononitrile with 2*H*-pyrido[1,2-*a*]pyrimidine-2,4(3*H*)-dione, and aryl aldehydes under the catalyzed, and microwave-irradiated conditions. The procedure reported by Mahmoud, and El-Saghier^127^ is a green protocol owing to the nontoxic effect of zinc oxide nanoparticles, and non-hygroscopic characters. Mainly, the structural nature of the substituents on the phenyl ring of the aldehyde affected the product yields since the nitro, chlorine, and fluorine substituents provided the highest yields (93–97%), while the electron-donating groups produced reduced yields of the products, specifically for *o*-amino substituent (45%). The nano-catalyst, zinc oxide nanoparticles, was prepared from a solution of zinc chloride in water with the addition of cyclohexane, ethyl alcohol, and dropwise addition of an aqueous ammonia solution (40%). As shown from the proposed mechanism in Scheme 40, the nano-catalyst acts as a coordinate, and Lewis acid in the activation of the carbonyl group of the aldehyde, and enable the enolization of the 2*H*-pyrido[1,2-*a*]pyrimidine-2,4(3*H*)-dione for the condensation step. The nano-catalyst also supported the cyclization of the pyran ring through an intramolecular nucleophilic attack of the carbonyl oxygen atom at the terminal nitrile group. Finally, [1,3]H migration gave the tricyclic products 51.

**Scheme 41:**
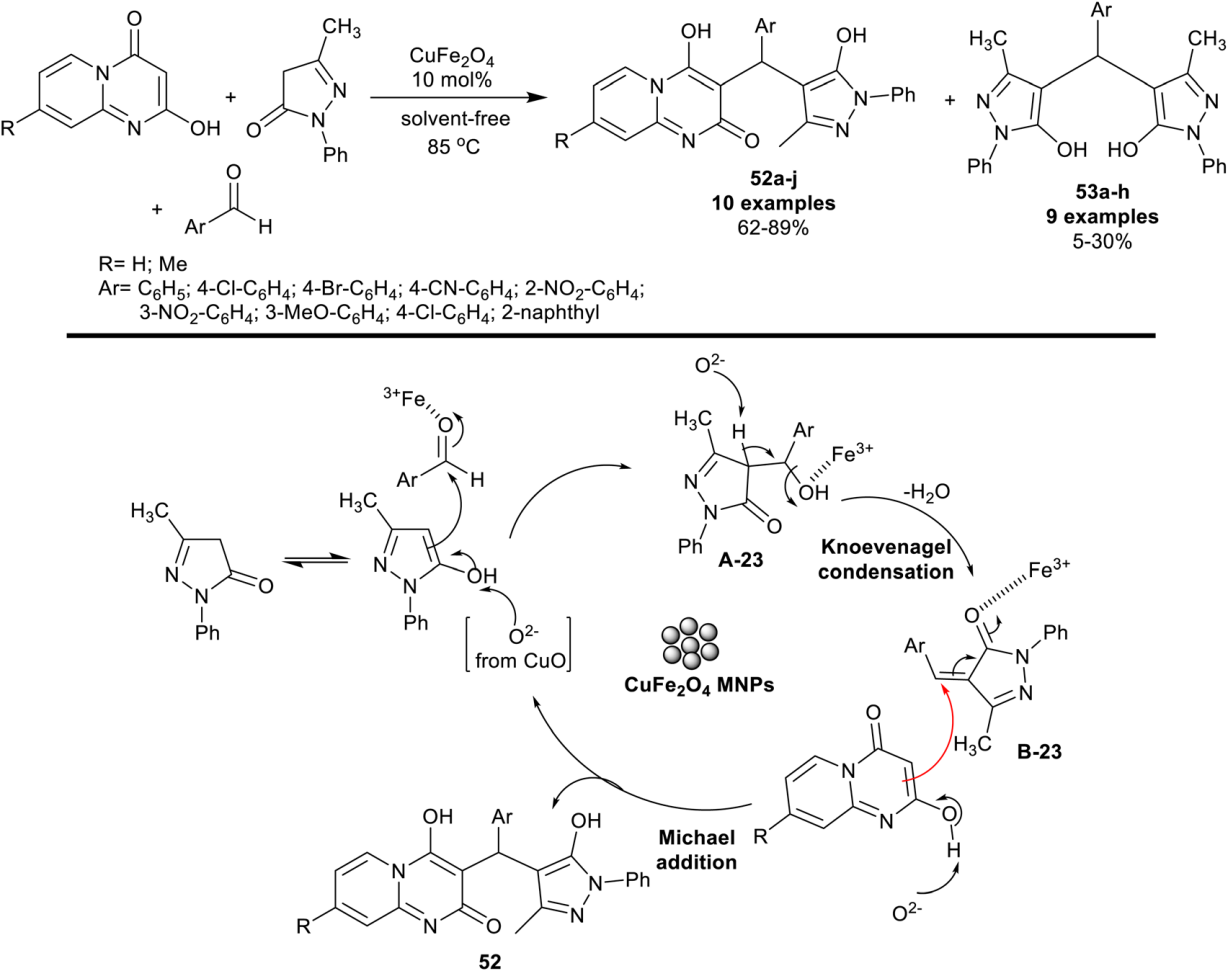
Synthesis of pyrazolylpyridopyrimidines.

Nano-catalysts such as CuFe_2_O_4_ are green catalysts, insensible for either air or moisture,^128–130^ and have been employed for the synthesis of varied heterocycles, such as five-membered ring heterocycles with two heteroatoms,^131^ five-membered ring heterocycles with three heteroatoms,^132,133^ five-membered ring heterocycles with four heteroatoms,^134,135^ fused heterocycles,^136^ and applied in cross-coupling reactions.^137^ Jannati *et al.*^138^ have applied a green procedure for the three-component one-pot synthesis of pyrazolylpyrido[1,2-*a*]pyrimidines 52a–j. Thus, reactions of 2-hydroxy-4*H*-pyrido[1,2-*a*]pyrimidine-4-ones with aryl aldehydes and 3-methyl-1-phenyl-1*H*-pyrazol-5-one under nano-catalytic conditions using CuFe_2_O_4_ gave the desired products 52a–j along with the formation of minor side products, bis(3-methyl-1-phenyl-1*H*-pyrazol-5-ol) 53a–h (Scheme 41). The nano-catalyst recyclability was achieved owing to the magnetic, and heterogeneous features of the nanocatalyst. Therefore, the simple preparation of the nano-catalyst and separation from the reaction mixture supported the green protocol and improve the product yield. Under solvent-free, and semi-scaled-up procedure conditions, product 52a was isolated from the reaction with 70% yield after two hours in the presence of the CuFe_2_O_4_ nano-catalyst. The plausible reaction mechanism was specified as a coordination bond and was formed between iron ions with the aldehydic carbonyl group, which acted as a Lewis acid to smoothly proceed the Knoevenagel condensation step with the active methylene group of the pyrazolone. The oxide included the metal oxide nanoparticles of the catalyst simplified the deprotonation step. Michael's addition step sequence took place between the pyrazolone intermediates, and 2-hydroxy-4*H*-pyridopyrimidineones to give the major products 52, while the minor products 53 were formed through the interaction of the arylidene intermediates with the pyrazolone substrate in its enol form.

## 4 Concluding remarks

Nanomaterials have been applied in diverse fields such as electronics, chemistry, medicinal and pharmaceutical fields owing to their excessive superficial area, volume ratios, conductivity, magnetic liability, and catalytic features. Specifically, magnetic nanoparticles have attracted the researchers' interest because of their remarkable appeals and prospective applications in various areas. Herein, different strategies for the synthesis of pyridopyrimidines under nano-catalytic conditions were highlighted. The nanocatalyst is efficiently applied in organic synthesis to improve the product yields and increase the rates of the reactions. The nanocatalyst delivered a high surface area of the nanoparticles, low costs, recyclability, ease of workup, and non-toxic features. Two main procedures were applied for the synthesis of the nanoparticles involving co-precipitation and sol–gel methods. Recently, we have reported the chemistry of pyridodipyrimidines, in which several synthetic approaches were achieved under nano-catalytic conditions.^139^ In particular, the varied methods for the preparation of pyrido[2,3-*d*]pyrimidines involved two-component reactions of aryl aldehydes with 6-amino-5-cyanoacetyl-2-thioxo-2,3-dihydropyrimidin-4(1*H*)-one or (2-aminopyridin-3-yl)methanol with aryl methanamines or 3-(aminomethyl)pyridin-2-amine with aryl methanethiols. The three-component synthesis involved the reactions of substituted amino-uracils with aryl aldehydes and activated nitriles, ketones, or cyanoacetyl heterocycles. The four-component reactions of thiobarbituric acid, aldehydes, ammonium acetate, and 4-hydroxy-2*H*-chromen-2-one gave the desired chromeno-pyrido[2,3-*d*]pyrimidines. The spirocyclic systems were synthesized from the reactions of 2-aminoethane-1-thiol hydrochloride with (2-nitroethene-1,1-diyl)bis(methylsulfane), isatins, and barbituric acid or from reactions of 2,6-diamino-pyrimidine-4(3*H*)-one or 6-aminouracil derivatives with isatins. On the other hand, pyrido[1,2-*a*]pyrimidines were synthesized from three-component reactions of 2-aminopyridines with aryl aldehydes, and active methylene components. The synthesis was extended through four-component reactions. In another route, analogs of pyrido[1,2-*a*]pyrimidines were prepared from reactions of 2*H*-pyrido[1,2-*a*]pyrimidine-2,4(3*H*)-dione with malononitrile, and aryl aldehydes or reactions of 2-hydroxy-4*H*-pyrido[1,2-*a*]pyrimidine-4-ones with aryl aldehydes and 3-methyl-1-phenyl-1*H*-pyrazol-5-one. The plausible mechanisms for each class of the desired compounds were drawn and described. The biological characteristics of the compounds as antimicrobial agents were also investigated.

## 5 Future prospective

Methods applied to prepare pyridopyrimidine compounds vary based on the nature of the reactants, using many innovative methods that aim to improve the reaction efficiency by increasing yields and rates. Consequently, nano-catalysts recently are being widely applied for the construction of compounds with a pyrimidine skeleton.^140–144^ Thus, consideration should be given to the productivity of the nano-catalyst relative to other catalysts, and the preparation of nano-catalysts at a lower cost. On the other hand, the ease of nanocatalyst preparation, and its separation from the reaction mixture to be recycled should be considered. Therefore, the recyclability of the nanocatalyst without losing its efficiency saves costs as well as re-preparation. Through the research discussed here, we found that research interest was directed toward improving the product yields and expanding the use and preparation of nanocatalysts, with little research devoted to studying the biological activities of these compounds. Therefore, the various identified biological activities of pyridopyrimidine compounds should be evaluated in future studies for the compounds that were prepared using nanocatalysts.

## 6 List of nanocatalysts

[γ-Fe_2_O_3_@HAp-SO_3_H]; Ni-doped TiO_2_ NPs; nano-Cu-doped TiO_2_; polycaprolactone NPs (PCL NPs); Fe_3_O_4_ MNPs; ZrO_2_ NPs; nano-MgO; SBA-15-Pr-SO_3_H; Fe_3_O_4_@SiO_2_@(CH_2_)_3_S–SO_3_H; Mn-ZIF-8@ZnTiO_3_; Fe_3_O_4_-ZnO-NH_2_-PW_12_O_40_; Fe_3_O_4_@TiO_2_@NH_2_@PMo_12_O_40_; aluminate sulfonic acid nanoparticles (ASA-NPs); nano-[Fe_3_O_4_@SiO_2_/*N*-propyl-1-(thiophen-2-yl) ethanimine][ZnCl_2_]; nano-Fe_3_O_4_@SiO_2_–SO_3_H; Fe_3_O_4_@NCs/Cu(ii); CTAB–water (admicellar system) containing nano-zinc oxide; Fe_3_O_4_@nano-cellulose/Ti(iv); [Fe_3_O_4_@ZrO_2_]; Fe_3_O_4_@SiO_2_-supported ionic liquid nanocatalyst; Fe_3_O_4_@FAp@Ni; Cu(ii)-PBABMD reserved on γ-Fe_2_O_3_@HAp magnetic core–shell; NiO NPs; nano-SiO_2_; nano-MnFe_2_O_4_; nano-Ni-doped TiO_2_; S8-NPs–SDS; ZnO-NPs; CuFe_2_O_4_.

## Ethical approval

The research does not comprise any human and/or animal studies and therefore does not necessitate the agreement of ethical committees, internal review boards, and ascertained guidelines.

## Abbreviations

γ-Fe_2_O_3_@SiO_2_: γ-Ferric oxide supported silicon dioxide
γ-Fe_2_O_3_@HAp-SO_3_H: HAp-encapsulated-γ-Fe_2_O_3_ supported sulfonic acid
TPP: Triphenylphosphine
dppf: Bis(diphenylphosphino)ferrocene
PVA surfactant: Poly(vinyl alcohol)
PCL: Polycaprolactone
TEBAC: Triethyl benzyl ammonium chloride
SBA-Pr-SO_3_H: Sulfonic acid functionalized nanoporous silica
ZnTiO_3_: Zinc titanate
ZIF-8: Zeolitic imidazolate framework-8
Fe_3_O_4_·PMO1: Fe_3_O_4_-ZnO-NH_2_-PW_12_O_40_
CTAB: Cetryltrimethyl ammonium bromide
UV-irradiation: Ultraviolet irradiation
SDS: Sodium dodecyl sulfate
NPs: Nanoparticles
Er-doped TiO_2_ NPs: Erbium-doped titanium dioxide nanoparticles
CuFe_2_O_4_: Copper ferrite

## Data availability

The datasets used are the accessible literature concerning the article theme.

## Conflicts of interest

The authors assert no conflicts of interest.

## Supplementary Material
